# Polysaccharide based biomaterials for advanced wound healing applications

**DOI:** 10.3389/fcimb.2026.1910532

**Published:** 2026-08-18

**Authors:** Pritiman Pothal, Sunny Chugh, Guramrit Kaur, Ramneet Kaur, Suraj Pratap Singh, Ashish Suttee, Pavitra Ranawat, Kamlesh Kumar, Ravi Pratap Barnwal, Gurpal Singh

**Affiliations:** 1 University Institute of Pharmaceutical Sciences, Panjab University, Chandigarh, India; 2 Department of Biophysics, Panjab University, Chandigarh, India; 3 Faculty of Applied Medical Sciences, Department of Pharmacognosy and Phytochemistry, Lovely Professional University (LPU), Phagwara, Punjab, India; 4 Department of Chemistry, University of Delhi, Delhi, India

**Keywords:** bioactive scaffolds, polysaccharide biomaterials, stimuli-responsive biomaterials, structure-function relationships, sustainable biomaterials, tissue regeneration, wound healing

## Abstract

Healing a wound is a complex biological process involving hemostasis, inflammation, cell proliferation, and tissue remodeling. Ad interim, polysaccharide based biomaterials have also attracted significant attention in wound healing due to their intrinsic biocompatibility, biodegradability and structural versatility, and ability to actively modulate the wound microenvironment. This review focuses on key polysaccharides, including alginate, chitosan and hyaluronic acid and discusses their roles across different stages of wound healing by correlating material structure with biological performance. The relationship between material structure and biological performance is discussed to understand their therapeutic effects. Recent advances in hybrid biomaterials, ion-coordination strategies, and stimuli-responsive dressings are highlighted for their roles in enhancing antimicrobial activity, promoting angiogenesis, and enabling controlled therapeutic delivery. Special emphasis is also placed on the emerging role of polysaccharide-based biomaterials in combating chronic wound-associated biofilms through disruption of the extracellular polymeric substance matrix, modulation of quorum-sensing pathways, and localized antimicrobial delivery. These multifunctional approaches improve infection control while simultaneously supporting tissue regeneration, thereby addressing one of the principal challenges associated with chronic wound management. The purpose of this review is to look at the impact of the use of these materials on the environment, specifically by looking at both their biodegradability and lessening of our dependency on synthetic polymers, as well as presenting an integrated design framework that links the composition, physicochemical characteristics, and biological demands of biomaterials within a time-based context to provide a rational approach for developing future wound dressings. Despite promising progress, challenges related to reproducibility, scalability, and clinical translation remain significant, underscoring the need for standardized evaluation and interdisciplinary approaches.

## 1 Introduction

**Figure 1:**
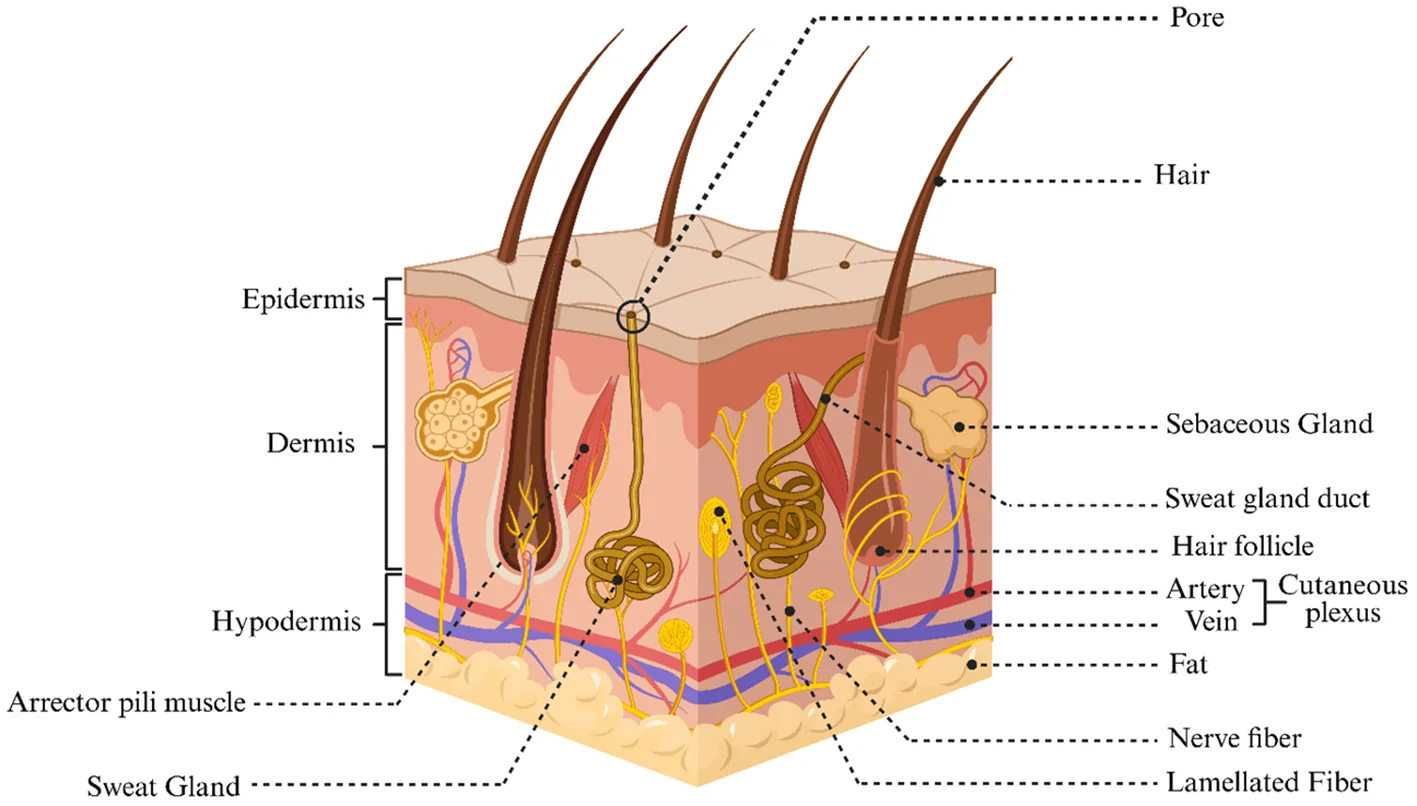
Structural organization of human skin showing a schematic cross-sectional view of the epidermis, dermis and hypodermis. The dermal layer contains vascular networks, nerve fibers, hair follicles and glandular structures. At the same time, the hypodermis is composed primarily of adipose tissue, which contributes to cushioning and insulation. This hierarchical architecture provides the biological foundation for tissue repair and wound healing processes.

The skin acts as a vital shield, safeguarding against physical harm, harmful microbes and excessive moisture loss, while preserving tissue integrity and the balance of the body. As the continuity of one’s skin is disrupted by an injury, surgical procedure, or long-term medical condition, the skin undergoes a series of processes and mechanisms to repair itself to a normal state as both an organ and a body part. The three major components of the skin are the outermost layer (epidermal), the middle layer (dermal) and the innermost layer of the body (hypodermis). Each layer works together to regulate the body’s immune response, heal wounds and provide protection (**Figure 1**).

Wound healing occurs through a series of overlapping biological stages, including hemostasis, inflammation, proliferation and remodeling, which are regulated by cytokines, growth factors, immune cells, and extracellular matrix dynamics (Cioce et al., 2024). Disruption of the above processes results in: delayed healing of wounds, the development of chronic wounds and the formation of abnormal scars. Chronic wounds are a significant burden for the worldwide healthcare system and commonly occur in diabetes patients, those with vascular disorders, older adults, and people with suppressed immune responses. In order for successful wound healing to take place, there must be adequate cellular infiltration into both the wound and surrounding tissues, and there must be an adequate blood supply to these areas so that there is an adequate deposition of new extracellular matrix proteins (Jin et al., 2025). Consequently, contemporary wound management has shifted toward biomaterial-based dressings capable of providing structural support while actively modulating the wound microenvironment through controlled moisture balance, antimicrobial activity, immunomodulation, and enhanced tissue regeneration. In clinical settings, chronic and complex wounds such as diabetic ulcers, pressure ulcers, and burn injuries remain difficult to manage due to persistent inflammation, infection risk, and impaired vascularization. These challenges highlight the need for advanced wound dressings that can actively support tissue repair while adapting to the dynamic wound microenvironment. A comparative summary of the functional roles of polysaccharide, protein, and hybrid biomaterials relevant to wound healing is presented in **Table 1**.

**Table 1:** Functional roles of polysaccharide, protein and hybrid biomaterials across different stages of wound healing.

Biomaterial	Key properties	Wound healing stage	Biological function	Clinical relevance	References
Alginate	High absorbency, gel-forming	Hemostasis, Inflammation	Exudate control, maintains moist environment	Useful in highly exuding wounds and burns	(Tahouri et al., 2026)
Chitosan	Antimicrobial, hemostatic	Hemostasis, Inflammation	Controls bleeding, reduces infection	Effective in infected and acute wounds	(Cassano et al., 2024)
Hyaluronic Acid	Hydrating, bioactive	Proliferation, Remodeling	Promotes cell migration, ECM formation	Supports tissue regeneration and reduces scarring	(Cassano et al., 2024)
Collagen (hybrid)	Structural protein	Proliferation	Supports cell adhesion and tissue growth	Enhances wound closure	(Geng et al., 2025)
Hybrid systems	Tunable properties	All stages	Controlled drug delivery, multifunctional support	Suitable for chronic and complex wounds	(Wang et al., 2026)

Conventional wound dressings, such as gauze, occlusive films, and foams, primarily provide physical protection but cannot actively regulate the wound microenvironment. In addition to clinical limitations, conventional synthetic wound dressings also contribute to increasing biomedical waste and long-term environmental burden due to their single-use nature and limited biodegradability. Growing awareness of sustainable healthcare practices has therefore encouraged the exploration of naturally derived biomaterials that can support effective wound healing while reducing ecological impact and improving material lifecycle management. Concerns about the environmental impact of wound management materials have received increasing attention, alongside the need to provide effective wound management in clinical practice. Traditional plastic and synthetic dressings contribute to a large volume of waste in the biomedical sector due to their difficulty breaking down (non-biodegradability) and their frequent use, creating a long-term negative environmental impact. Conversely, naturally derived polysaccharides (alginate, chitosan, hyaluronic acid) have the potential to provide biodegradable, sustainable materials for wound management; they can be easily degraded in the body (physiologically) and present a reduced burden on the environment. Developing ‘next generation’ wound dressings, with an emphasis on efficient use of materials and on addressing sustainability concerns, will remain the focus of the wound care materials industry (Gupta et al., 2025).

The traditional “one-size fits all” approach to wound care has not been effective. The field is now moving toward the creation of custom wound dressings tailored to the individual physiological characteristics of each wound and its healing stage. Custom wound dressings are increasingly made from biomaterials derived from natural macromolecules, including polysaccharides (e.g., alginate, chitosan, hyaluronic acid) and proteins (e.g., collagen, silk fibroin, gelatin), due to their biological compatibility, biodegradability, and natural activity that supports cellular function (Patrocinio et al., 2023). Among natural macromolecules, alginate, chitosan and hyaluronic acid, collectively referred to here as ACH polysaccharides, represent a versatile class of bioactive materials for wound management. Alginate provides effective moisture retention and exudate control; chitosan contributes antimicrobial and hemostatic properties and hyaluronic acid promotes cell migration and extracellular matrix remodeling. The combination of these polymers enables synergistic modulation of inflammation, angiogenesis and tissue regeneration (Tahouri et al., 2026). Furthermore, their biodegradability and renewable sourcing make these materials attractive candidates for developing wound care technologies that balance therapeutic effectiveness with environmental sustainability. There is a growing need to develop customized wound care solutions and, as such, the use of natural macromolecular biomaterials is now considered central to future wound-healing solutions.

**Figure 2:**
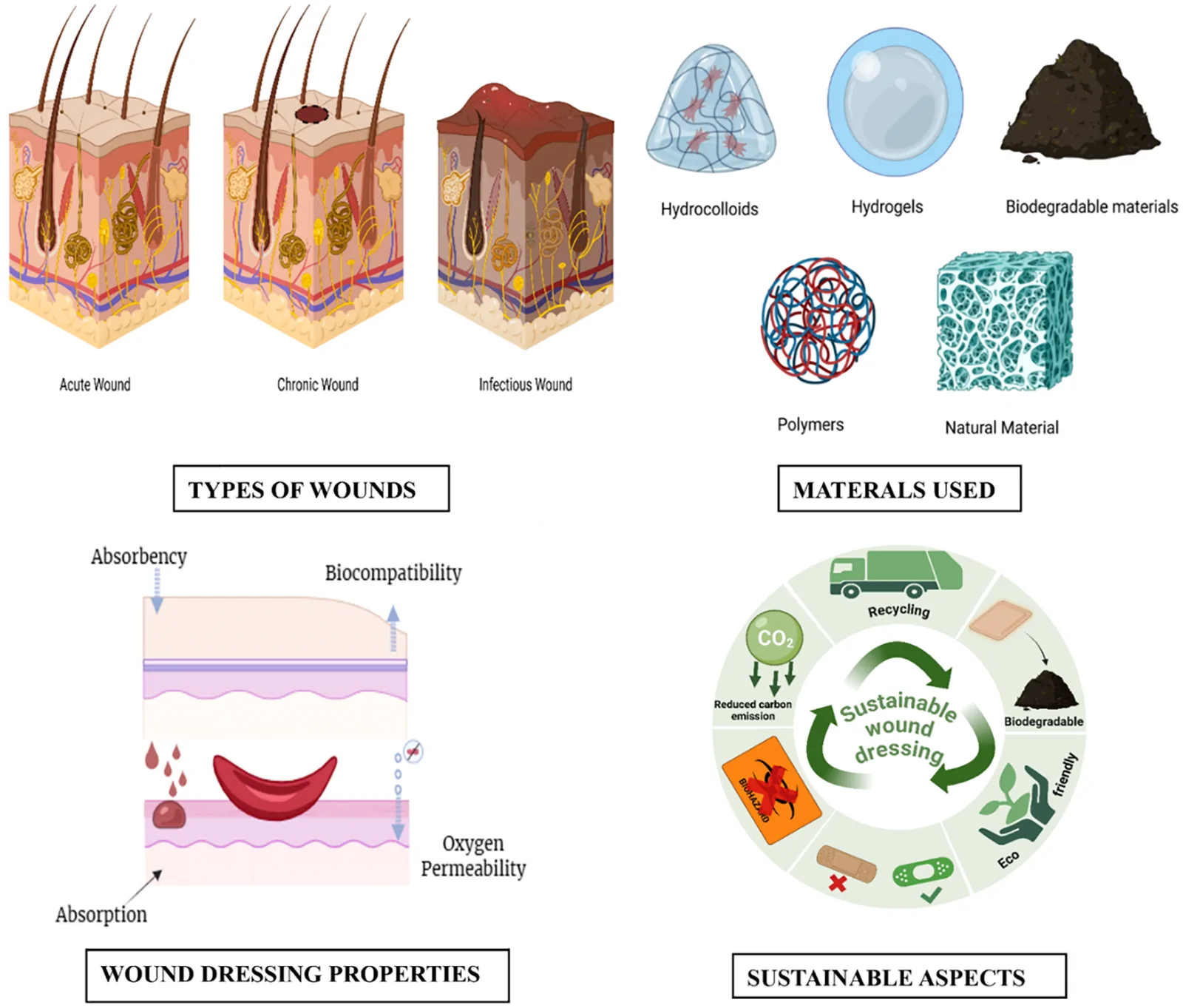
Conceptual framework illustrating key considerations in the design of advanced wound dressings. The upper-left panel presents representative wound types, including acute, chronic, and infectious wounds. The upper-right panel highlights the major classes of dressings, including hydrogels, polymeric systems, and biodegradable biomaterials. The lower left panel summarizes essential functional properties including exudate absorption, oxygen permeability and biocompatibility. The lower-right panel emphasizes sustainability-related factors such as biodegradability, recyclability, and environmental impact. Together, these elements provide an integrated perspective for the rational development of next-generation wound management systems.

Despite considerable advances in wound dressing technologies, chronic wound-associated biofilms remain one of the principal barriers to successful tissue repair and clinical recovery. Biofilms are structured microbial communities enclosed within a self-produced extracellular polymeric substance (EPS) matrix composed primarily of polysaccharides, proteins, extracellular DNA, and lipids. This protective matrix enhances microbial persistence by limiting antimicrobial penetration, reducing immune clearance, and facilitating coordinated bacterial survival through quorum-sensing mechanisms. Consequently, biofilm-associated infections contribute to prolonged inflammation, impaired angiogenesis, delayed re-epithelialization, and reduced responsiveness to conventional antimicrobial therapies. These challenges have stimulated growing interest in multifunctional polysaccharide-based biomaterials capable of simultaneously disrupting biofilm architecture, enhancing localized antimicrobial delivery, modulating inflammatory responses, and promoting tissue regeneration. Integrating biofilm-targeted therapeutic strategies with advanced biomaterial design therefore represents a critical direction for the development of next-generation wound dressings for chronic and infected wounds (Almuhanna, 2025). Unlike conventional reviews that describe biomaterials individually, this review integrates polysaccharide chemistry, scaffold architecture, coordination interactions, and biological function within the context of stage-specific wound healing. By correlating physicochemical properties with molecular healing mechanisms and clinical requirements, the review provides a comprehensive framework to support the rational design and translation of next-generation wound dressings. It emphasizes how material properties can be aligned with the dynamic needs of the wound microenvironment to improve therapeutic outcomes. In addition, the review highlights clinically relevant considerations, including infection control, exudate management, and tissue regeneration, to bridge the gap between material design and practical wound care applications. The overall conceptual framework adopted in this review is illustrated in **Figure 2**, which integrates polysaccharide chemistry, scaffold design, biofilm regulation, and stage-specific biological functions to guide the rational development of advanced wound dressings.

## 2 Literature search strategy

This review was conducted as a narrative review to provide a comprehensive overview of recent advances in polysaccharide-based biomaterials for wound healing. Unlike a systematic review or meta-analysis, the objective was to critically synthesize current knowledge, highlight mechanistic insights, and identify emerging trends in biomaterial design, biological performance, and clinical translation.

A comprehensive literature search was performed using Scopus, Web of Science, PubMed, and Google Scholar to identify relevant peer-reviewed publications published between 2010 and 2026. Searches were conducted using combinations of Boolean operators (AND/OR) with keywords including “polysaccharide biomaterials,” “wound healing,” “alginate,” “chitosan,” “hyaluronic acid,” “hydrogels,” “nanofibers,” “biodegradable dressings,” “biofilms,” “chronic wounds,” “metal-ion coordination,” “tissue regeneration,” and “smart wound dressings.” The literature selection prioritized original research articles and high-quality review papers published in English that investigated the design, physicochemical characteristics, biological functions, antimicrobial activity, biofilm interactions, coordination chemistry, and clinical applications of polysaccharide-based biomaterials for wound management. Landmark studies that established the foundation of the field were retained, while recent high-impact publications (particularly from 2023-2026) were incorporated to ensure that the review reflects current scientific advances.

Publications lacking sufficient mechanistic detail, studies unrelated to wound healing biomaterials, conference abstracts, editorials, duplicate reports, and non-English articles were excluded. Following retrieval, titles and abstracts were screened for relevance, and the full texts of potentially eligible articles were critically evaluated based on their scientific quality, methodological rigor, novelty, and contribution to understanding structure function relationships and therapeutic performance. Reference lists of key publications were also examined to identify additional relevant studies. The selected literature was subsequently synthesized to provide an integrated perspective on the relationship between polysaccharide chemistry, scaffold engineering, biofilm management, and stage-specific wound healing.

## 3 Phases of wound healing

Wound healing is a highly coordinated and dynamic biological process that restores tissue integrity following injury through a sequence of overlapping yet distinct phases, namely hemostasis, inflammation, proliferation, and remodeling. Successful tissue repair depends on the precise temporal and spatial regulation of cellular responses, cytokines, growth factors, and extracellular matrix remodeling. Disruption of any of these tightly regulated events may result in delayed healing, chronic wound formation, or excessive scar development. Hemostasis is the body’s immediate response to tissue damage. It stops bleeding and starts the healing process. When blood vessels are injured, a chain of events begins that causes them to become smaller, platelets to stick together and, finally, a stable fibrin clot to form. This clot serves as a temporary structure, preventing blood loss and attracting inflammatory cells by releasing signaling molecules, including fibronectin and platelet-derived growth factor (PDGF). The fibrin network is the first structure to hold cytokines, including interleukin-1 (IL-1), IL-6 and tumor necrosis factor-alpha (TNF-α) and it lays the foundation for the next step in healing (Zhang et al., 2026). In addition to preventing blood loss, the provisional fibrin matrix functions as a temporary extracellular scaffold that facilitates inflammatory cell recruitment and supports the initiation of tissue repair. Within hours of an injury, inflammation begins and is critical for eliminating microbial contamination and clearing damaged tissue. Neutrophils are some of the first cells to respond. They move toward the wound region in response to chemotactic signals such as TNF-α and transforming growth factor beta (TGF-β). These cells eliminate pathogens and remove dead tissue by generating reactive oxygen species (ROS) and enzymes that break down proteins. Monocytes enter the region and differentiate into macrophages. These cells not only eat up dead cells but also release vital growth factors, such as VEGF and basic fibroblast growth factor (bFGF), which help tissues heal (Yang et al., 2025). At this point, the interaction between DAMPs and PAMPs is very important for coordinating the immune response. However, persistent inflammation resulting from prolonged microbial colonization and biofilm formation may disrupt the normal transition to the proliferative phase, thereby contributing to chronic wound development and impaired tissue regeneration (Singh et al., 2025). Wounds have been shown to positively affect health by eliminating harm and supporting the restoration of health. In addition, all factors listed below contribute significantly to proliferation: re-epithelialization, new blood vessels, fibroblast proliferation and the formation of extracellular matrix (ECM). Keratinocytes at the wound margins proliferate and migrate into the defect to fill the space. Among the factors involved in the process, two examples are epidermal growth factor (EGF) and transforming growth factor alpha (TGF α). The action of transforming growth factor beta (TGF β) also leads to the production of collagen, glycosaminoglycans, and proteoglycan matrix by activated fibroblasts. Granulation tissue supports cell movement and provides a substrate for the development of new (Singh et al., 2025) blood vessels; this occurs through the deposition of matrix components by fibroblasts. One of the hallmark events of this phase is angiogenesis, largely driven by the vascular endothelial growth factor (VEGF), which increases the formation of new blood vessels to meet the oxygen and nutrient demands of the regenerating tissue. Macrophages and endothelial cells collaborate with fibroblasts to establish a provisional ECM that fosters cell adhesion and tissue maturation. Successful completion of the proliferative phase is essential for restoring vascularized granulation tissue and accelerating wound closure, thereby reducing the risk of chronic wound progression. Maturation and remodeling represent the final and longest stage of wound healing, potentially spanning weeks to years depending on wound severity. As healing progresses from the early to later phases, fibroblasts reorganize collagen matrices by replacing type III collagen with the stronger type I collagen, restoring tensile strength and function to the tissue. Closure of an open wound occurs by the contraction of myofibroblasts and matrix metalloproteinases (MMPs) mediate the degradation and remodeling of collagen. The tissue will regain approximately 50-80% of its initial tensile strength at various times after an injury. However, complete recovery to pre-injury levels is rarely achieved. There is a predictable sequence of biological events throughout wound healing, but several factors influence it locally and systemically. The nature, duration and intensity of many of these factors can have positive or negative effects on tissue function and repair. Understanding how these factors influence the healing process is essential for designing effective wound treatment regimens tailored to each patient. **Table 2** presents key local and systemic factors that affect wound healing. Collectively, these tightly coordinated biological events provide the molecular foundation for successful tissue repair. However, chronic wounds are frequently characterized by persistent inflammation, impaired angiogenesis, excessive protease activity, and microbial biofilm formation, all of which interfere with the normal healing cascade. Consequently, considerable research has focused on the development of multifunctional polysaccharide-based biomaterials capable of simultaneously regulating these biological pathways while providing structural support, antimicrobial activity, immunomodulation, and controlled therapeutic delivery. The molecular mechanisms underlying these biomaterial mediated effects are discussed in the following section.

**Table 2:** Factors affecting the wound healing process locally and systemically.

Factors	Effect on wound	References
Oxygen levels	Oxygen is essential for cellular metabolism and energy production. Appropriate oxygen levels promote cell function, angiogenesis and collagen synthesis. Hypoxia can either speed up or slow down healing depending on its duration. Chronic hypoxia negatively affects the process of wound healing.	(Dong and Wang, 2023)
Infection	Infection can delay wound healing by increasing inflammation, degrading growth factors and facilitating bacterial biofilm formation, which hinders healing.	(Mamun et al., 2024)
Age	Age-related changes in cellular regeneration, slower inflammatory responses and reduced collagen production all delay wound healing.	(Wilkinson and Hardman, 2023)
Hormones (Estrogen and Androgens)	The hormones estrogen and androgen regulate the expression of genes involved in wound healing, inflammation and tissue regeneration.	(Mamun et al., 2024)
Stress (Glucocorticoids)	Stress-linked glucocorticoids weaken immune responses, reduce pro-inflammatory cytokines and impair immune cell function, thereby delaying healing.	(Kolimi et al., 2022)
Diabetes	Diabetes slows wound healing by lowering oxygen levels, increasing ROS, elevating MMP levels and reducing angiogenesis, which can delay healing or even lead to amputations.	(Las Heras et al., 2024)
Obesity	Obesity makes it more likely for wounds to get infected and limits the flow of blood to the cut. Bioactive adipokines secreted by adipose tissue and macrophages interfere with tissue repair.	(Frasca and Strbo, 2022)
Alcohol consumption	Alcohol impairs immunological response, inhibits angiogenesis and increases the wound’s susceptibility to infection, delaying recovery.	(Kolimi et al., 2022)
Smoking	Smoking constricts blood vessels, reducing oxygen flow and interferes with the healing process by lowering oxygen intake, which is required for tissue regeneration.	(Wilkinson and Hardman, 2023)

### 3.1 Molecular regulation of wound healing by polysaccharide based biomaterials

Successful wound healing is regulated by a complex molecular network involving cytokines, growth factors, ECM remodeling and cell matrix interactions. Beyond serving as passive wound coverings, polysaccharide-based biomaterials actively influence these molecular pathways by modulating inflammatory signaling, promoting angiogenesis, regulating oxidative stress, facilitating extracellular matrix remodeling and enhancing cellular communication. These biomaterial-mediated interactions provide the mechanistic basis for designing advanced wound dressings capable of restoring tissue homeostasis while overcoming the pathological barriers associated with chronic and infected wounds. Accordingly, the rational design of advanced wound dressings requires precise integration of biomaterial composition, physicochemical properties, and molecular signaling pathways. By tailoring polysaccharide chemistry, scaffold architecture, coordination interactions, and bioactive functionalization, modern wound dressings can actively regulate key biological processes involved in tissue repair while addressing the pathological features of chronic wounds. Coordination chemistry has emerged as an important strategy for enhancing the biological performance of polysaccharide-based biomaterials by regulating their physicochemical properties, therapeutic stability, and interactions with cellular signaling pathways. The incorporation of bioactive metal ions into polysaccharide matrices enables simultaneous modulation of scaffold integrity, antimicrobial activity, angiogenesis, immunomodulation, and controlled therapeutic delivery, thereby improving the functional performance of advanced wound dressings. Nevertheless, the incorporation of inorganic nanocarriers and metal-based therapeutic systems also necessitates careful evaluation of their immunometabolic effects and long-term biosafety, as nanomaterial physicochemical characteristics can influence macrophage polarization, inflammatory signaling, oxidative stress, and host immune responses. Rational nanocarrier design should therefore integrate both therapeutic efficacy and immunological safety to facilitate successful clinical translation (Sahu et al., 2026). ACH composite matrices coordinated with bioactive metal ions such as Zn²^+^, Ca²^+^, Cu²^+^ and Ag^+^ exhibit synergistic antibacterial, angiogenic, and immunomodulatory activities that enhance both infection control and tissue regeneration. For example, coordination of Zn²^+^ and Cu²^+^ with hydroxyl and carboxyl functional groups within polysaccharide matrices can stabilize bioactive molecules, prolong VEGF-mediated angiogenic signaling and promote endothelial cell proliferation, thereby facilitating neovascularization during tissue repair. Similarly, Ca²^+^-mediated crosslinking within alginate and protein based matrices enhances scaffold stability, promotes integrin-mediated cell adhesion, and modulates Smad2/3 signaling, thereby supporting extracellular matrix remodeling while reducing excessive fibrosis. Furthermore, modulation of HIF-1α stability by selected bioactive metal ions promotes angiogenesis under hypoxic wound conditions, thereby supporting vascular regeneration in chronic wounds (Liu et al., 2026). Collectively, these mechanisms demonstrate that coordination chemistry provides a versatile platform for integrating scaffold design with molecular regulation, enabling the development of multifunctional polysaccharide-based wound dressings capable of simultaneously promoting tissue regeneration, controlling infection, and improving clinical healing outcomes (**Table 3**) (Wang et al., 2024; Chen et al., 2025; Khulood et al., 2025; Mo et al., 2025; Ni et al., 2025; Zhang et al., 2025).

**Table 3:** Molecular biology mechanisms in wound healing and the role of biomaterials with coordination chemistry strategies.

Healing stage	Key molecules/pathways	Biomaterial strategies (proteins & polysaccharides)	Role	References
Hemostasis	PDGF, TGF-β, Fibrin clot	Collagen scaffolds stabilize the clot, gelatin sponges support platelet adhesion.	Ca²^+^ crosslinking strengthens alginate gels and coordination sites bind clotting factors.	(Moosazadeh Moghaddam et al., 2023; Cui et al., 2023)
Inflammation	IL-1, IL-6, TNF-α, VEGF	Chitosan stimulates macrophage activation and alginate maintains a moist wound bed.	Cationic biopolymers coordinate with immune cell membranes; Zn²^+^ and Ag^+^ ions modulate cytokine release and antimicrobial activity.	(Li et al., 2024)
Proliferation	VEGF, bFGF, EGF and ECM deposition	Hyaluronic acid hydrogels enhance angiogenesis and silk fibroin supports fibroblast migration.	Cu²^+^, Zn²^+^ and Mn²^+^ ions coordinate with growth factor receptors, stimulating angiogenesis and collagen synthesis.	(Li et al., 2024)
Remodeling	MMPs, TGF-β, Collagen I/III maturation	Silk fibroin modulates TGF-β signaling and collagen scaffolds promote tensile strength.	Metal-ligand coordination networks stabilize scaffold structure, Ca²^+^/Mg²^+^ regulate ECM mineralization and remodeling.	(Zhao et al., 2024)

### 3.2 Coordination chemistry mechanisms in polysaccharide based wound dressings

The therapeutic effect of metal coordinated polysaccharide dressings depends on the coordination interactions that occur between the metal ions and the functional groups that are present in the natural polysaccharides such as the carboxyl groups (-COO^-^) of alginate and hyaluronic acid and the amine (-NH_2_) and hydroxyl (-OH) groups of chitosan. The formation of reversible coordination bonds through this interaction produces cross-linked three-dimensional networks in hydrogels that concentrate the mechanical stability while maintaining sufficient flexibility of the dressings for use in wounds (Cui et al., 2022). The degree of coordination directly influences degradation behavior by the regulating crosslink density and network architecture. Higher coordination density generally slows hydrolytic and enzymatic degradation, prolonging structural integrity and allowing sustained therapeutic function, whereas weaker coordination promotes faster biodegradation and tissue remodeling. Such tunable degradation is mainly advantageous for chronic infected wounds, where prolonged dressing stability is often required. Metal coordination also contributes to the controlled retention and release of bioactive molecules. In a coordinated network, diffusion of encapsulated drugs and growth factors occur slowly hence limiting burst release and achieving a sustained local delivery of regenerative mediators such as VEGF, PDGF and bFGF. This prolonged bioavailability promotes angiogenesis, fibroblast proliferation, collagen deposition and re-epithelialization throughout the healing process. In particular, coordinated metal ions have a direct impact on the biological activities. Zinc ions aid in the process of epithelial regeneration while at the same time help in collagen production (Zhuang et al., 2026). Copper ions help in the formation of new blood vessels *via* the HIF-1α/VEGF pathway and are also known for their antiseptic properties. Silver ions, on the other hand, provide a wide range of anti-bacterial and anti-biofilm properties. Coordination chemistry, therefore, not only enhances the physicochemical properties of polysaccharide dressings but also provides the properties of controlled degradation, sustained release of therapeutics agents as well as wide biological applications of the polysaccharide based formulations (**Table 4**).

**Table 4:** Quantitative comparison of native and metal-coordinated polysaccharide dressings in preclinical wound healing studies.

Polysaccharide	Coordinating metal	Wound model	Key *in vitro* findings	Key *in vivo* findings	Proposed mechanism	References
Alginate	Ag^+^	MRSA/S. aureus infected wound	>99% antibacterial activity, enhanced hydrogel stability	Faster wound closure, reduced bacterial burden	Antibacterial activity + sustained Ag^+^ release	(Shi et al., 2023)
Alginate	Zn²^+^	Diabetic wound	Increased fibroblast viability, improved compressive strength	Enhanced collagen deposition and angiogenesis	Zn²^+^-mediated ECM remodeling and epithelial regeneration	(Wang et al., 2025)
Chitosan	Cu²^+^	Full-thickness infected wound	Improved endothelial cell migration	Increased VEGF/CD31 expression and neovascularization	HIF-1α/VEGF activation	(Hou et al., 2022)
Chitosan	Ag^+^	Biofilm wound	Strong anti-biofilm activity	Reduced inflammation and bacterial colonization	Membrane disruption and ROS generation	(Tang et al., 2026)
Hyaluronic acid	Fe³^+^	Chronic wound	Improved hydrogel elasticity and sustained drug release	Accelerated tissue remodeling	Dynamic coordination crosslinking	(Tang et al., 2026)
Hyaluronic acid	Zn²^+^	Diabetic wound	Enhanced keratinocyte proliferation	Increased re-epithelialization	Controlled Zn²^+^ release and immune regulation	(Wang et al., 2025)

## 4 Types of wounds and requirements for wound dressings

**Figure 3:**
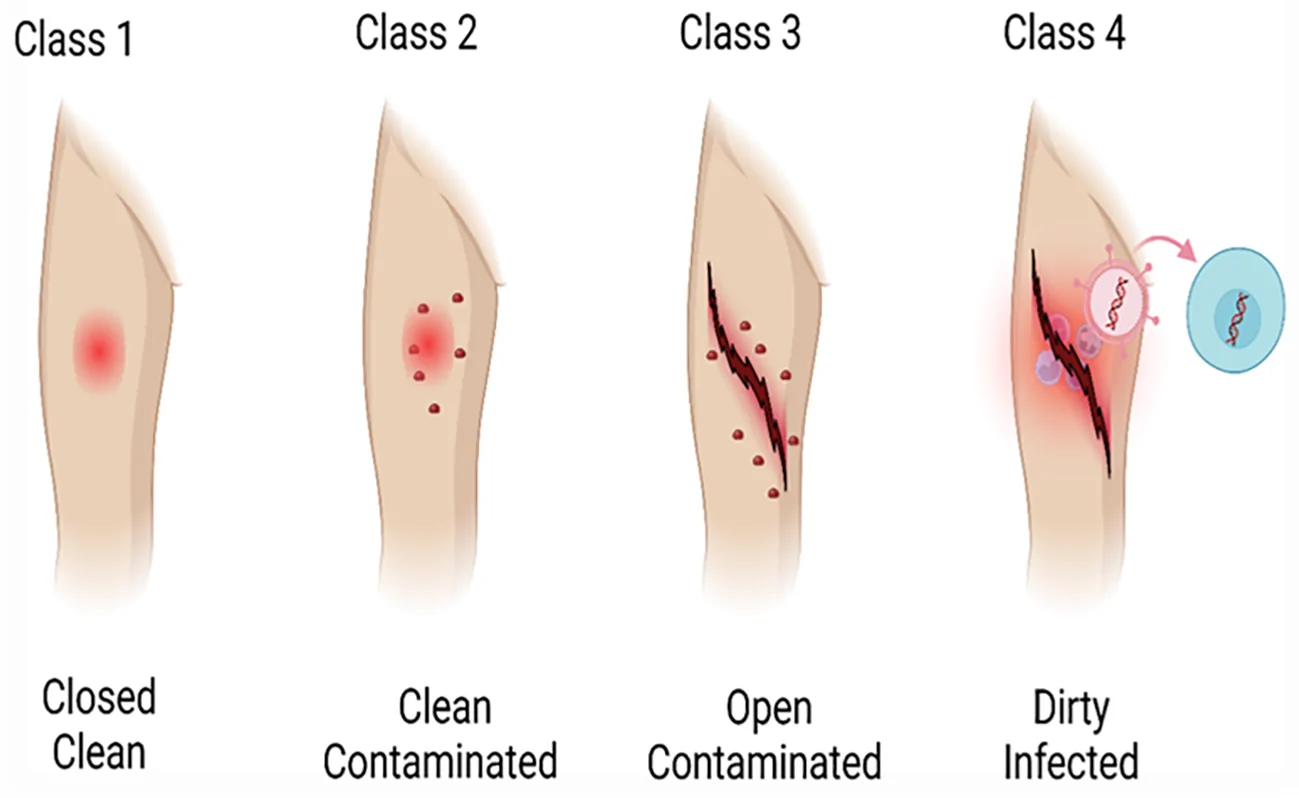
Schematic representation of classification of wounds according to the degree of microbial contamination. Class I (clean) wounds have minimal infection risk, whereas Class II (clean-contaminated) and Class III (contaminated) wounds exhibit progressively increasing microbial exposure. Class IV (dirty/infected) wounds contain established infection and biofilm formation, leading to persistent inflammation and delayed wound healing.

Wounds are changes in the structure and function of the skin and the tissues that support it. They can result from trauma, infection, surgery, or long-term problems. Correct wound classification is critical for making therapeutic decisions and selecting the best dressing technique. The Centers for Disease Control and Prevention (CDC) classify wounds into four types, as depicted in **Figure 3**, based on the number of germs they contain: clean wounds (Class 1), clean-contaminated (Class 2), contaminated (Class 3) and dirty-infected (Class 4) (Herman et al., 2023). This classification is based on whether the wound was contaminated by internal organs or invaded by germs due to trauma. In a more practical therapeutic setting, wounds are typically classified as acute or chronic based on the time required to heal and the biological processes underlying them. Surgery, mechanical injuries and minor cuts are the most prevalent causes of acute wounds. Acute wounds heal rapidly and predictably, typically resolving within days to weeks. They demonstrate clear tissue injury and contain minimal bacterial colonization. Unlike acute wounds, chronic wounds fail to progress through the normal stages of healing and remain continually inflamed for prolonged periods. Some common examples of chronic wounds include pressure sores, diabetic ulcers (foot), venous ulcers (legs) and ischemic ulcers. All of these conditions result from underlying diseases such as diabetes, immobility, venous insufficiency, or peripheral artery disease. Chronic wounds are often associated with excessive moisture production, biofilm formation, active inflammation, impaired formation of new blood vessels, and loss of the extracellular matrix (Yang et al., 2024). Diabetic foot ulcers can be difficult to treat due to problems with the feet’s blood vessels and nerves. These pathological alterations impair normal tissue repair and increase susceptibility to infection. Venous leg ulcers are caused by poor blood flow. They are most common on the lower legs, leading to edema, fibrin formation, and the presence of microorganisms. When there is not enough blood flow to an area, and therefore not enough oxygen and nutrients are delivered, that can create ischemic wounds. Ischemic wounds are often made up of dry, dead tissue. If you are unable to move, you are at greater risk of developing pressure ulcers (also called bed sores or decubitus ulcers). Pressure ulcers develop when constant pressure on bone areas cuts off blood flow and damages tissue. Puncture wounds created from a sharp object or an animal bite, in general, are extremely difficult to treat because they may have a foreign body lodged within them and are prone to developing polymicrobial infections. Burns can be caused by heat, chemicals, or electrical sources and can vary in severity; therefore, burns require specialized dressings that keep the area moist, prevent infection, and support the wound-healing process (Witkowski et al., 2024). Dressings used on wounds have to be flexible enough to cater to the unique needs of each type of wound produced as a result of injury. Optimal dressings will control moisture, prevent contamination from progressing to infection, maintain pH levels, allow gas exchange with the external environment and provide a protective layer over the wound. In addition, they will promote cellular migration and the formation of new blood vessels. Dressings for wounds should also make it an easier and less painful or uncomfortable experience to apply or remove from the skin; this is especially true for fragile or long-term wounds. Early-Generation Dressings do not always meet all of these criteria. Therefore, the move is away from these types of materials toward physiologically active and responsive materials. For example, biologically derived natural macromolecular biomaterials are particularly attractive because they have demonstrated high biocompatibility with living systems, a continuum of biodeterioration and intrinsic physiological characteristics. These materials typically mimic the body’s natural extracellular matrix, thereby affording cell attachment and can be designed to deliver therapeutic agents to the wound environment or to respond to variations in the biological conditions of the wound microenvironment. The use of this type of material will allow designers to create advanced dressings compatible with the care and management of both acute and chronic wounds, as well as those that may present different needs at different points in time.

### 4.1 Role of biofilms in chronic wounds and therapeutic opportunities for polysaccharide biomaterials

Chronic wounds represent a major clinical challenge because they frequently become colonized by microbial biofilms, which are now recognized as one of the principal causes of impaired wound healing and persistent infection. Unlike planktonic microorganisms, bacteria within biofilms exist as highly organized multicellular communities that irreversibly adhere to the wound surface and exhibit markedly enhanced tolerance to host immune defenses and antimicrobial therapies. The prevalence of mature polymicrobial biofilms is particularly high in chronic wounds, including diabetic foot ulcers, venous leg ulcers, and pressure ulcers, where they contribute to prolonged inflammation, delayed re-epithelialization, impaired angiogenesis, excessive protease activity, and extracellular matrix degradation. Consequently, biofilm-associated infections interfere with the orderly progression of the wound-healing cascade, preventing successful transition from the inflammatory phase to tissue regeneration. These challenges highlight the need for multifunctional therapeutic strategies capable of simultaneously disrupting biofilm architecture, controlling microbial burden and promoting tissue repair (Guerrero-Rodriguez et al., 2026; Rothmaier et al., 2026). A defining characteristic of microbial biofilms is the formation of a self-produced EPS matrix, which provides structural integrity and protection to microbial communities. The EPS matrix is primarily composed of extracellular polysaccharides, proteins, extracellular DNA (eDNA), lipids, and water, forming a highly hydrated three-dimensional network that anchors microorganisms to the wound surface. This protective barrier limits the penetration of antimicrobial agents, reduces phagocytic clearance by immune cells, and creates localized physicochemical gradients that facilitate microbial persistence. Furthermore, the EPS matrix functions as a reservoir for enzymes, toxins, and signaling molecules that collectively promote chronic inflammation and tissue destruction. As a result, conventional antimicrobial therapy often fails to eradicate mature biofilms despite demonstrating efficacy against planktonic bacteria. Therefore, disruption of the EPS matrix has emerged as a critical therapeutic objective in the development of advanced wound dressings for chronic wound management (Zhao et al., 2023; Azeem et al., 2025).

Biofilm development is further regulated by quorum sensing, a cell-density-dependent communication mechanism through which bacteria coordinate gene expression by producing and detecting diffusible signaling molecules. Quorum sensing controls multiple aspects of biofilm biology, including microbial adhesion, extracellular matrix production, virulence factor secretion, metabolic adaptation, and biofilm dispersal. These coordinated responses significantly enhance bacterial survival under hostile conditions while contributing to antimicrobial tolerance and recurrent wound infection. Persistent quorum-sensing activity also sustains inflammatory cytokine production, impairs macrophage polarization toward the pro-regenerative M2 phenotype, and disrupts growth factor signaling, thereby delaying tissue regeneration. Consequently, therapeutic approaches capable of interfering with quorum-sensing pathways while simultaneously promoting tissue repair have attracted increasing attention in contemporary wound-healing research (Zeng et al., 2023; Xu et al., 2026). Polysaccharide-based biomaterials provide a versatile platform for addressing these complex biological challenges because they combine excellent biocompatibility with intrinsic biological activity and extensive opportunities for functional modification. Chitosan exhibits broad-spectrum antimicrobial activity through electrostatic interactions between its positively charged amino groups and negatively charged bacterial cell membranes, resulting in membrane destabilization, increased permeability, and disruption of biofilm architecture. In addition, chitosan has been reported to interfere with EPS organization, enhance immune cell activation, and promote macrophage polarization toward a regenerative phenotype. Alginate-based hydrogels contribute by maintaining a moist wound environment while serving as efficient carriers for antimicrobial agents, metal ions, enzymes, and nanoparticles capable of penetrating the biofilm matrix and providing sustained localized therapeutic delivery. Hyaluronic acid supports wound healing by facilitating cell migration, extracellular matrix remodeling, and angiogenesis while simultaneously reducing bacterial adhesion through hydration-mediated surface effects. Similarly, bacterial cellulose provides a mechanically robust and highly hydrated scaffold that supports localized drug delivery and prolonged antimicrobial activity. Recent advances in multifunctional polysaccharide composites further integrate coordination chemistry, metallic nanoparticles, antimicrobial peptides, and stimuli-responsive release systems to achieve simultaneous biofilm disruption, infection control, immunomodulation, angiogenesis, and tissue regeneration (Ikrar et al., 2026). Recent advances have further expanded the capabilities of polysaccharide-based wound dressings through the development of intelligent and stimuli-responsive platforms that dynamically respond to changes in the wound microenvironment. These systems are designed to release antimicrobial agents, growth factors, or anti-inflammatory therapeutics in response to local stimuli such as acidic pH, elevated ROS, bacterial enzymes, or increased glucose concentrations characteristic of chronic diabetic wounds. The incorporation of coordination chemistry, metallic nanoparticles, antimicrobial peptides, and enzyme-responsive linkers further enhances localized therapeutic delivery while minimizing systemic toxicity. Such multifunctional platforms simultaneously disrupt biofilm architecture, regulate inflammation, promote angiogenesis, and accelerate tissue regeneration, thereby providing a promising strategy for personalized management of chronic biofilm-associated wound infections (Dhar et al., 2026). The anti-biofilm performance of advanced wound dressings is commonly evaluated using complementary microbiological and imaging techniques that characterize both bacterial viability and biofilm architecture. Crystal violet staining is widely employed to quantify total biofilm biomass, whereas colony-forming unit (CFU) assays determine bacterial viability following treatment. Confocal laser scanning microscopy combined with fluorescent live/dead staining enables three-dimensional visualization of biofilm thickness, structural integrity, and bacterial viability within intact biofilms. Scanning electron microscopy provides detailed morphological characterization of bacterial attachment and extracellular matrix disruption, while fluorescence-based molecular assays can further assess quorum-sensing activity and extracellular DNA distribution. Integration of these analytical approaches provides comprehensive evaluation of the anti-biofilm efficacy of newly developed wound dressings and facilitates comparison between different biomaterial platforms (Haval et al., 2025). Collectively, these findings demonstrate that effective management of chronic wounds requires biomaterials capable of simultaneously regulating tissue regeneration and controlling biofilm-associated infections. Future research is expected to focus on multifunctional polysaccharide-based wound dressings incorporating smart drug-delivery systems, coordination chemistry, antimicrobial peptides, bioactive nanoparticles, and stimuli-responsive hydrogels capable of adapting to the dynamic wound microenvironment (Shi et al., 2025; Ferraz, 2026). Future investigations should also prioritize standardized *in vitro* and *in vivo* biofilm models, quantitative evaluation of biofilm disruption using confocal laser scanning microscopy, colony-forming unit analysis, and biofilm biomass assays, together with long-term safety assessments of metal-ion-coordinated biomaterials. Such standardized evaluation will facilitate comparison across studies and accelerate clinical translation of next-generation wound dressings. The integration of biofilm-targeted therapeutic strategies with advanced polysaccharide engineering therefore represents a promising direction for the development of next-generation wound dressings that combine enhanced antimicrobial performance with improved clinical healing outcomes (**Table 5**).

**Table 5:** Major polysaccharides used in wound dressings and their applications in acute and chronic infected wounds.

Polysaccharide	Key physicochemical properties	Primary role in wound healing	Preferred application in infected wounds	Representative examples	Ref.
Alginate	Highly hydrophilic, excellent exudate absorption, Ca²^+^-mediated gelation	Hemostasis, moisture retention, exudate management	**Acute infected wounds:** traumatic wounds, surgical wounds, burns with heavy exudate. **Chronic infected wounds:** diabetic foot ulcers and venous leg ulcers when combined with Ag^+^, Zn²^+^ or Cu²^+^ for enhanced antibiofilm activity and tissue regeneration.	Calcium alginate, Ag-alginate hydrogel, Zn-alginate composite	(Liu et al., 2024; Do et al., 2025; Pourmadadi et al., 2025)
Chitosan	Cationic, biodegradable, intrinsic antimicrobial and hemostatic properties	Hemostasis, antimicrobial activity, immune regulation	**Acute infected wounds:** contaminated lacerations and surgical wounds requiring rapid bacterial control. **Chronic infected wounds:** diabetic ulcers and biofilm associated wounds using metal ion or nanoparticle-modified chitosan systems.	Chitosan hydrogel, Cu-chitosan, Ag-chitosan dressing	(Nomura-Contreras et al., 2026)
Hyaluronic acid	Highly biocompatible, hydrophilic, ECM component	Cell migration, angiogenesis, ECM remodeling	**Acute infected wounds:** promotes re-epithelialization and tissue repair. **Chronic infected wounds:** chemically modified or metal coordinated hydrogels for prolonged inflammation control and sustained therapeutic delivery.	HA hydrogel, Zn-HA hydrogel	(Shang et al., 2026)
Bacterial cellulose	High tensile strength, excellent water retention	Moist wound environment, mechanical protection	**Acute infected wounds:** superficial wounds and burns. **Chronic infected wounds:** multifunctional composites loaded with antimicrobial agents or metal nanoparticles for biofilm inhibition.	BC-Ag, BC-ZnO	(Shang et al., 2026, Psarrou et al., 2023)
Dextran	Hydrophilic, easily functionalized	Drug delivery and controlled release	Mainly chronic infected wounds requiring sustained antimicrobial or growth factor delivery.	Oxidized dextran hydrogel	(Yasmin et al., 2026)
Hybrid polysaccharide systems	Synergistic mechanical and biological properties	Multifunctional wound repair	Suitable for both acute and chronic infected wounds, particularly complex biofilm associated wounds requiring simultaneous antimicrobial, immunomodulatory and regenerative functions.	Alginate-chitosan, Alginate-HA, Chitosan-cellulose composites	(Liu et al., 2024)

Bold values indicate the most significant applications as within each comparison category.

## 5 Overview of natural macromolecules in wound care

**Figure 4:**
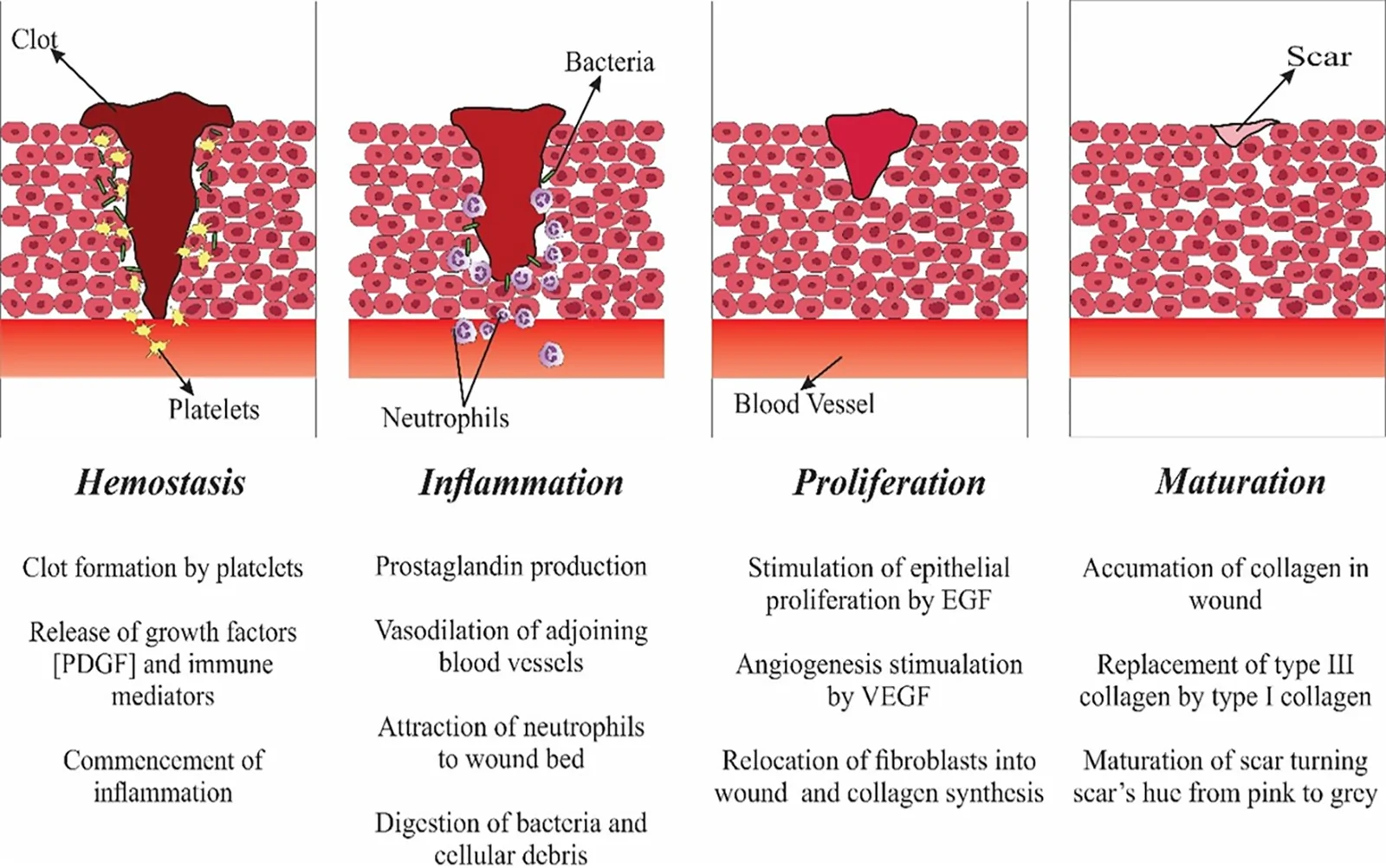
Schematic representation of the four overlapping phases of cutaneous wound healing: hemostasis, inflammation, proliferation and maturation (remodeling). Hemostasis involves platelet aggregation and fibrin clot formation, providing a provisional matrix and releasing growth factors such as PDGF and TGF-β. The inflammatory phase is characterized by the recruitment of neutrophils and macrophages to eliminate microbial contamination and cellular debris. During proliferation, keratinocyte migration, fibroblast activation, extracellular matrix deposition, and angiogenesis, driven by VEGF and EGF, contribute to granulation tissue formation and wound closure. The maturation phase involves collagen remodeling, the replacement of type III collagen with type I collagen, and the restoration of tissue tensile strength through scar formation. Understanding these coordinated biological events provides a foundation for designing biomaterial-based wound dressings that can modulate healing outcomes.

Natural macromolecules such as polysaccharides are increasingly recognized for their role in contemporary wound care because they share structural features with components of the body’s extracellular matrix and may therefore be compatible with biological systems and possess biological activity. Such polymers typically derive from plants, marine organisms, animals and microbes and consist mainly of polysaccharides or proteins that protect and support the repair of the body’s tissues. Due to their nature, natural macromolecules interact directly not only with cellular processes but also with other biomolecular processes involved in hemostasis, inflammation, proliferation and remodeling (Yang et al., 2023; Zhao et al., 2023; Arunim et al., 2024). **Figure 4** shows a schematic illustration of the different cell and molecular events that occur during the four classic stages of wound healing. The interactions described allow for changes in some aspects of the immune response, as well as recruiting cells to the area and repairing the extracellular matrix, which are all critical for rapid wound healing. Alginate is one of the most studied polysaccharides for treating wounds and is derived from brown seaweed. When it contacts wound exudate, it exchanges calcium ions (which form a gel when mixed with sodium ions) with sodium ions from the wound fluid. Thus, the formed gel allows for cellular migration, maintains a moist environment around the wound and facilitates hemostasis (life-saving) regarding bleeding wounds (Abourehab et al., 2022). Zinc and calcium-alginate formulations have demonstrated superior clotting ability, making them suitable for trauma-related and post-surgical applications (Ponsen et al., 2022). Alginate’s ability to be used with human tissue, its long gel time, and its capacity to serve as a scaffold for both fibroblasts and keratinocytes all contribute to its significant potential in regenerative therapies for wounds. Chitosan and chitin also exhibit many attractive characteristics, such as bioadhesive, hemostatic, and antimicrobial properties. Chitosan is obtained from the exoskeletons of various crustaceans (e.g., crabs) or the cell walls of various fungi; it promotes clot formation, inhibits microbial growth, and enhances collagen production. High-molecular-weight chitosan can form viscous gels, retain fluids, and interact with proteins at the wound site. Water-soluble chitin-derived products can also promote collagen deposition and reepithelialization, making them ideally suited for use in diabetic ulcers and chronic wounds. Composite dressings containing chitosan are currently being investigated to determine whether they can serve as vehicles for delivering growth factors, antibiotics, or even gene-based therapeutics to promote the healing of deep tissue wounds (Naveedunissa et al., 2025). Collagen, the most abundant structural protein in mammals, plays a significant role in wound healing. As a key ECM component, collagen supports cellular adhesion, chemotaxis and new tissue formation. Its low immunogenicity and natural degradation by matrix metalloproteinases make it an ideal substrate for full-thickness wound applications (Zhu et al., 2024).

Collagen scaffolds, sponges and powders were found to enhance granulation, epithelialization and vascularity of healing tissue in both laboratory and clinical settings. Novel applications of collagen-based hydrogels loaded with mesenchymal stem cells are attracting interest as a means to promote faster wound healing and create long-lasting changes in the underlying structure of the healed area (Alberts et al., 2025a). Organisms like Acetobacter Xylinum produce bacterial cellulose, a pure, crystalline, and incredibly fine form of cellulosic material. The advantageous physicochemical properties of bacterial cellulose, including its high water-retention capacity, mechanical strength, and excellent biocompatibility, make it an attractive material for advanced wound dressings. Bacterial cellulose can also provide a “moist” barrier for the wound while still allowing oxygen to pass through. It will not stick to the wound surface, making it easier to remove and less traumatic when changing the dressing over time. The large surface area and porosity of bacterial cellulose enable the addition of therapeutic agents, such as antimicrobial and anti-inflammatory medications. Another significant type of bioactive molecule that is sulfated polysaccharides derived from brown algae is fucoidan, which has demonstrated its ability to exert several different effects, including, but not limited to, being an antioxidant, having anti-inflammatory properties, having pro-angiogenic properties, possessing antimicrobial properties, and reducing inflammatory responses due to lipopolysaccharide stimulation as well as collagen degradation from degradation due to fibroblasts. It is thought that hydrogels or hybrid materials containing fucoidan will enhance wound healing in dermal burns and diabetic wounds. In addition to providing a structural framework for tissue formation, fucoidan may act as a biological mediator, enhancing fibroblast migration, stimulating angiogenesis, and increasing cytokine production. Silk sericin, derived from the cocoon of *Bombyx mori*, is rich in amino acids and functional groups like carboxyl and hydroxyl moieties. It has demonstrated significant efficacy in burn wound healing because it helps fibroblasts develop, produces collagen, and helps wounds close (Esmail et al., 2026).

**Figure 5:**
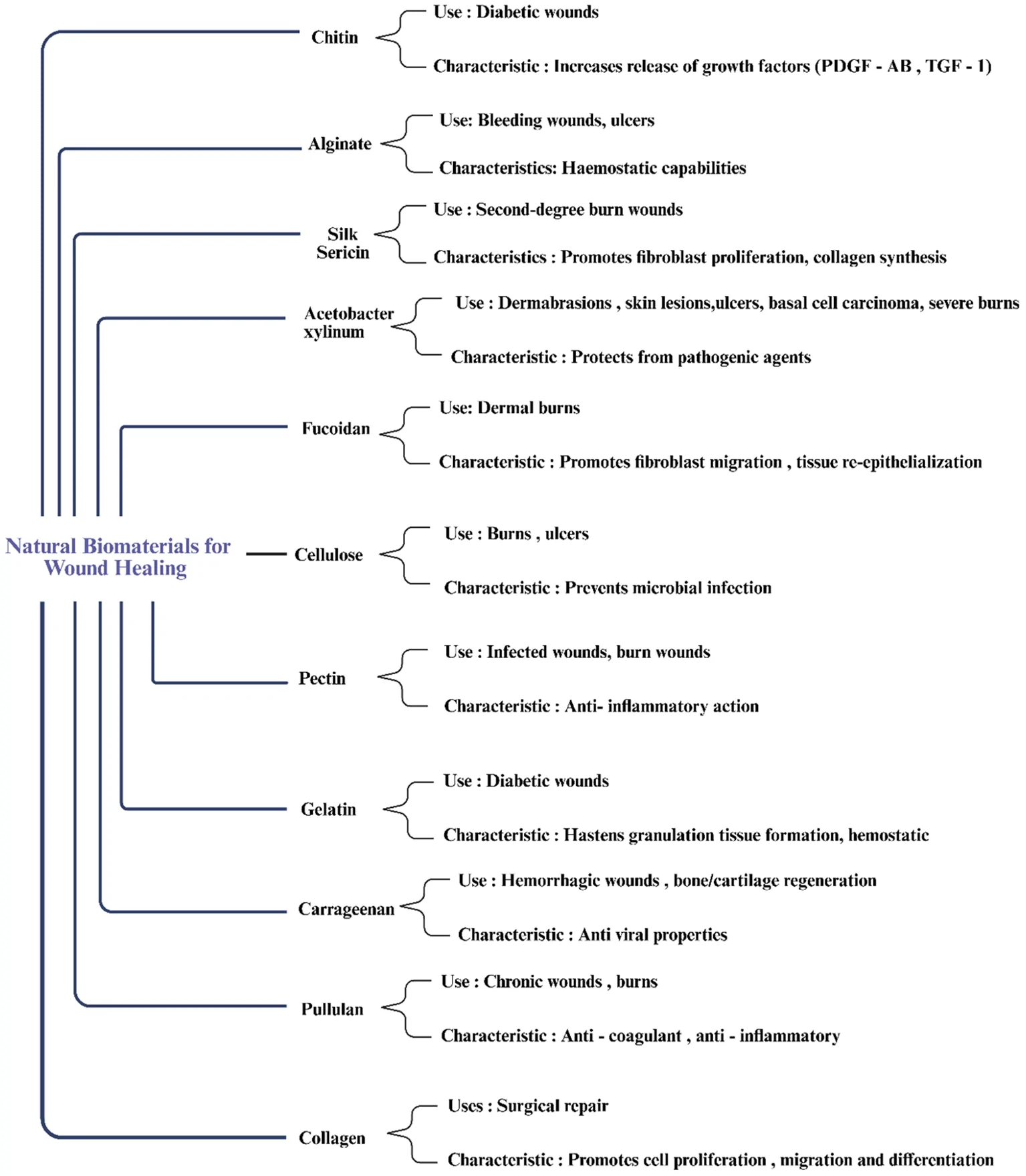
Overview of representative natural biomaterials employed in wound healing applications, including chitin, alginate, silk sericin, bacterial cellulose (Acetobacter xylinum), fucoidan, cellulose, pectin, gelatin, carrageenan, pullulan and collagen. The diagram highlights their typical clinical or therapeutic applications, such as the management of diabetic wounds, burns, ulcers, and hemorrhagic injuries, along with key functional attributes, including hemostatic activity, antimicrobial protection, anti-inflammatory effects, and promotion of fibroblast proliferation and tissue re-epithelialization. These biomaterials contribute to different stages of wound repair through structural support, modulation of the wound microenvironment and enhancement of regenerative cellular responses.

Materials made from silk break down naturally, are safe for individuals and work well with skin, making them ideal for sensitive wounds. Other notable biopolymers are gelatin, carrageenan, pectin and pullulan. Gelatin is a type of collagen that has been broken down by water. It makes strong films and holds onto medicines effectively; thus, it is great for getting active ingredients to the wound bed. Carrageenan and pectin are polysaccharides that come from plants. They help lower inflammation and make gels. Because of these traits, they are better at healing infected or leaking wounds (Liu et al., 2024). Pullulan can be produced by fermenting certain types of fungus and is useful for blocking air in the product (to provide flexibility), creating a film, etc. However, because it usually requires additional assistance to function properly in wound care, it has limited benefit. Other examples of natural macromolecules not only protect against illness and dehydration but also contribute to the healing process by providing chemical signals to cells and by interacting with them. These macromolecules are available in many forms (film, fiber, hydrogel, sponge, and composite) and can be used to treat wounds of varying shapes, sizes, depths, and exudate levels. This characteristic allows these biomolecules to address the needs of today’s wound care industry. These biomolecules will form the basis for the development of new types of wound dressings, as more patients and caregivers seek dressings that are biocompatible, durable and capable of performing multiple functions. A general overview of commonly studied natural biomacromolecules, their target wound types and their biological roles is illustrated in **Figure 5**.

Polysaccharides and proteins are often combined in hybrid dressings. However, since both polysaccharide and protein based materials offer distinct attributes, they can be compared in detail on an individual basis. For example, collagen, gelatin, and silk fibroin are all proteins with various attributes that provide structural support and bioactive signaling, as well as cell-adhesion sites that replicate those found in the extracellular matrix. Together, these three characteristics support angiogenesis, fibroblast migration, and tissue remodeling, making them primarily suitable for very deep surgical wounds or mechanically stressed tissue. However, proteins may exhibit batch-to-batch variability, limited mechanical stability, and susceptibility to enzymatic degradation, which can reduce long-term durability (Sun et al., 2023). By contrast, polysaccharides such as alginate, chitosan, and hyaluronic acid are highly effective for moisture retention, hemostasis, and antibacterial activity. Their ability to form hydrogels or ionically crosslinked networks makes them especially advantageous for exudative wounds and ulcers. However, polysaccharides often lack inherent bioactive signaling and many require chemical modification to achieve appropriate mechanical strength. Hybrid protein-polysaccharide systems combine the tensile strength and bioactivity of proteins with the hydration and hemostatic properties of polysaccharides. These systems have demonstrated a synergistic effect as indicated by increased angiogenesis (VEGF-mediated) and ECM remodeling. However, there are challenges regarding stability, reproducibility and the approval process of these techniques. When all of these aspects are considered and organized into an approach tailored to the type of wound, it becomes apparent that protein-based scaffolds are best suited for use under surgical and mechanical loads, while polysaccharide-based hydrogels are effective for treating ulcers with excess moisture (exudate). At the same time, hybrid protein-polysaccharide materials offer innovative options for treating chronic and infected wounds that require multiple functions. This comparison, which summarizes the unique benefits and drawbacks of each type of biomaterial in **Table 6**, creates a major deficiency in specificity, as identified in prior literature.

**Table 6:** Comparative overview of proteins, polysaccharides, and protein-polysaccharide hybrid biomaterials in wound healing: key advantages, limitations, clinical applications, and coordination chemistry and nanotechnology based enhancements.

Type of biomaterial	Key advantages	Key disadvantages	Optimal wound applications	Coordination or nanotech enhancement	References
Proteins (Collagen, Gelatin, Silk Fibroin)	Strong tensile strength, natural ECM mimicry, and bioactive signaling promote angiogenesis.	Enzymatic degradation, variability, and poor long-term stability	Surgical wounds, mechanically stressed wounds	Metal-ion reinforcement (Ca²^+^, Fe³^+^) improves stability and integrin signaling.	(Rodionova et al., 2025)
Polysaccharides (Alginate, Chitosan, Hyaluronic Acid)	Excellent moisture retention, hemostasis, antibacterial action, and hydrogel formation	Weak mechanical strength, limited bioactivity	Exudative ulcers, burns, shallow wounds	Zn²^+^/Cu²^+^ coordination enhances angiogenesis; nanoparticles add antimicrobial strength	(Amponsah et al., 2025)
Protein-Polysaccharide Hybrids	Synergistic ECM mimicry + hydration promotes angiogenesis and antimicrobial performance.	Stability and reproducibility issues, regulatory challenges	Chronic wounds, infected wounds requiring multifunctional scaffolds	Nanoparticle composites and dual-ion coordination (Zn²^+^/Ag^+^, Cu²^+^) yield multifunctionality	(Quan et al., 2025; Ma et al., 2025; Moradifar et al., 2025)

**Figure 6:**
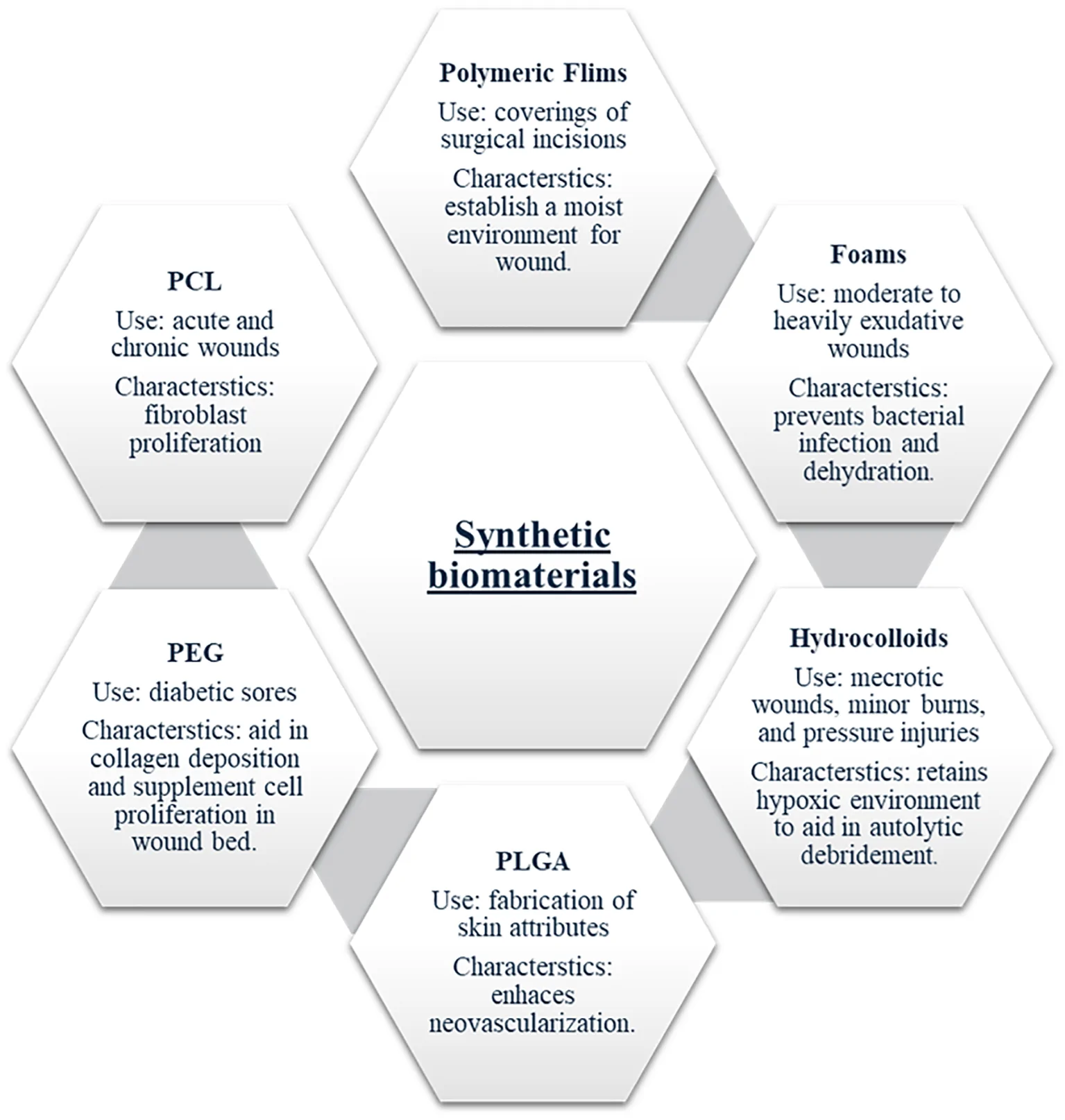
Overview of commonly used synthetic biomaterials and dressing platforms in wound management, including hydrocolloids, foams, polymeric films, polycaprolactone (PCL), polyethylene glycol (PEG) and poly(lactic-co-glycolic acid) (PLGA). These materials provide distinct therapeutic functions, such as maintaining a moist wound environment, absorbing exudate, providing structural support, and promoting cellular proliferation and neovascularization. Synthetic biomaterials are widely employed, either alone or in combination with natural macromolecules, to enhance mechanical stability, controlled drug delivery, and overall healing performance in acute and chronic wounds.

Compared to protein-based scaffolds, polysaccharide-based systems exhibit superior moisture retention and hemostatic properties but relatively lower mechanical strength, highlighting the importance of hybrid material design for multifunctional wound applications. Polymers derived from nature offer several biological advantages. However, synthetic biomaterials are also widely favored due to their ability to be produced in various sizes and possess customizable mechanical features, as depicted in **Figure 6**.

## 6 Macromolecular dressings and their forms and delivery strategies

**Figure 7:**
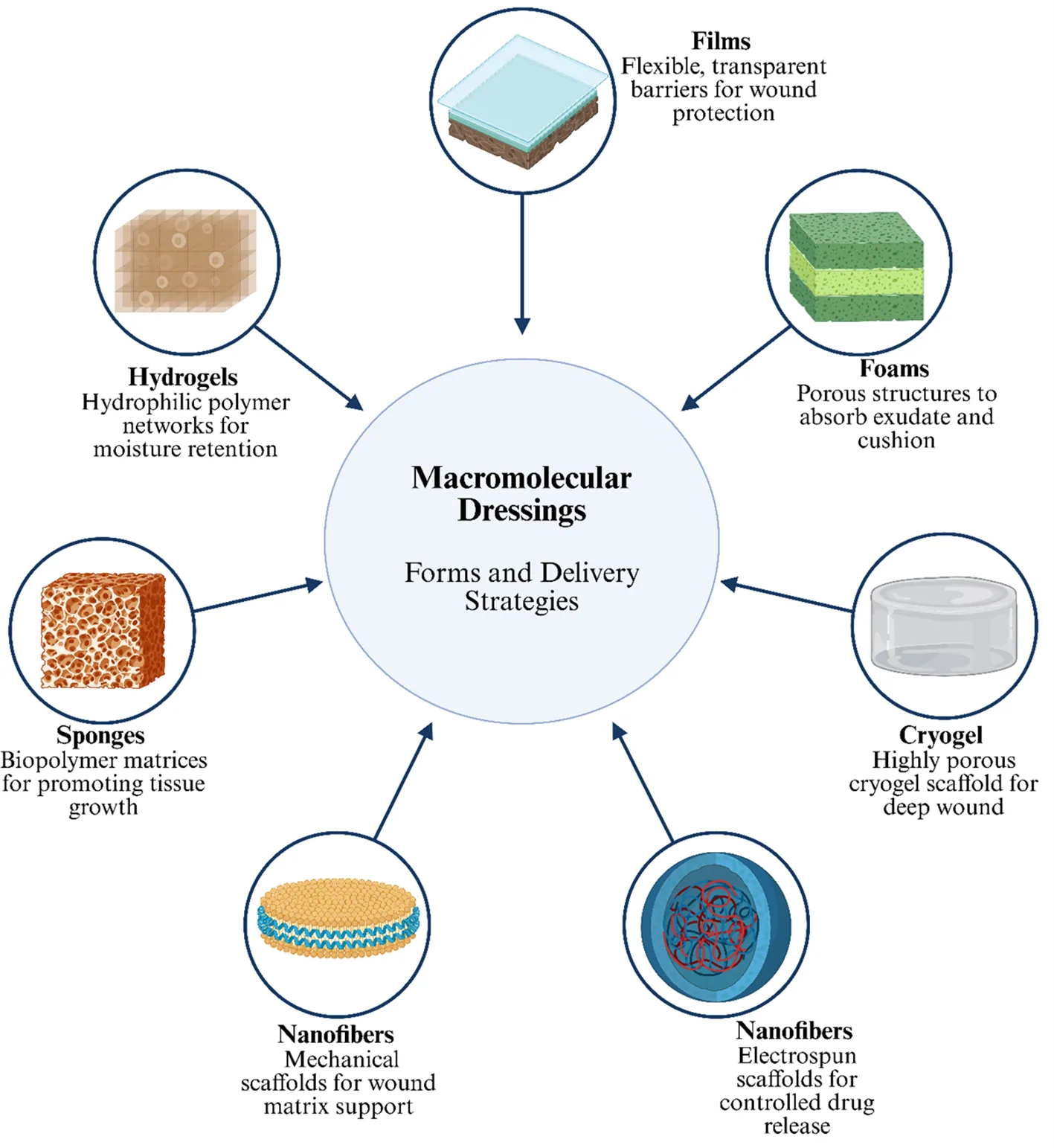
Representative macromolecular wound dressing platforms illustrating diverse structural formats and delivery functionalities. Hydrogels provide hydrated polymeric networks that support moisture retention and bioactive diffusion, while polymeric films function as flexible protective barriers. Foams and sponges offer porous architectures for exudate absorption and mechanical cushioning. In contrast, cryogels enable highly interconnected scaffolds suitable for deep or irregular wounds. Nanofibrous systems, including electrospun matrices, provide extracellular matrix–mimetic structures that facilitate cellular infiltration, tissue support, and controlled therapeutic release. Collectively, these macromolecular dressing formats enable tailored wound management strategies by modulating the local healing microenvironment.

Hydrogels and foams based on ACH are valuable models for assessing polysaccharide wound dressings in terms of water retention and biocompatibility, while utilizing natural polysaccharides allows for substantial flexibility in designing numerous wound dressing formulations with application-specific structural and functional characteristics. The polymeric structure of these formulations provides mechanical stability and an ideal moist environment for wound healing. Therefore, these dressings serve a dual purpose by providing adequate wound coverage and facilitating the healing process when applied. Many products are made from formulations of these materials, including hydrogels, films, foams, sponges, nanofibers and cryogels (Thang et al., 2023; Gounden and Singh, 2024). An illustrated summary of the major forms of macromolecular dressings and their primary roles in wound healing is presented in **Figure 7**. All are designed to treat a specific type of injury at a particular stage of treatment. Due to their composition, natural polymers offer researchers a range of structural/physicochemical properties for developing dressings that mimic extracellular matrices, control moisture levels, and enhance cells’ ability to migrate, proliferate, and form new blood vessels. Hydrogels provide a hydrated, three-dimensional polymeric network that maintains moisture balance, facilitates oxygen diffusion, and supports cellular migration and proliferation within the wound microenvironment. To facilitate tissue repair, macromolecules such as alginate, hyaluronic acid, chitosan, and collagen create microenvironments. These microenvironments interact with cells and bioactive substances. All of the materials investigated demonstrated excellent stability, enabling accurate administration of drugs, growth hormones and stem cells. The ability of hydrogels to encapsulate medicinal chemicals enables the drugs to be released in a controlled and sustained manner. This not only enhances the body’s natural healing process but also lessens the impact of adverse effects on the system (Parvin et al., 2024; Zhang et al., 2024). Recent advancements in hydrogel technology have enabled the precise release of therapeutic compounds in response to specific conditions in wounds. These parameters include pH levels, temperature variations or enzymatic activity. Biopolymer dressings are useful for treating minor abrasions and lacerations. These delicate, ethereal layers not only prevent the area from becoming infected or physically damaged but also help retain moisture in the wound. The maintenance of epithelial cells’ hydration and the enhancement of their motility are both dependent on the presence of their films. Enhancing oxygen transport throughout the vascular network is one way they encourage the development of new blood vessels. Utilizing materials that exhibit flexibility, transparency, and oxygen impermeability, such as gelatin, cellulose derivatives, and pullulan, is one way to improve film compositions (Sheokand et al., 2023). Transparent coatings can provide insight into the wound-healing process. This significant advantage suggests that the dressing will need to be changed less frequently, thereby increasing the patient’s comfort. To treat wounds with substantial exudate, foam dressings are used (Alberts et al., 2025b). Polyurethane or a mixture of organic and synthetic polymers is considered to be the most common substance used in the production of foams. Despite retaining greater moisture, they continue to provide the vital healing hydration required for effectiveness. The outer layer’s ability to facilitate the movement of certain molecules while simultaneously preventing the entry of germs contributes to the internal regulation of temperature and humidity. As a result, an environment is created that supports enzymes and cellular processes functioning at their highest potential. In addition to being an excellent method for reducing pressure ulcers, foam structures are also believed to be safe for use after surgery due to their cushioning properties. The incorporation of silver nanoparticles or iodine into foam matrices significantly enhances their antibacterial activity. As a result, these foam matrices are suited for the treatment of wounds susceptible to or at increased risk of infection (Nguyen et al., 2023). A design that improves the circulation of air and water is incorporated into sponge dressings made from biomaterials such as collagen, pectin, and chitosan. The identified ingredients create an environment conducive to granulation tissue formation, thereby enhancing cellular migration and the generation of new blood vessels. Because of their malleable nature and ability to conform to irregularly shaped injuries, they are particularly effective for treating complex trauma or surgical wounds. It is possible to improve sponges by adding hemostatic agents or growth factors to facilitate coagulation and support tissue repair. Recent advancements in composite sponges have demonstrated their effectiveness in treating diabetic foot ulcers and surgical site infections. They do this by enabling re-epithelialization and reducing inflammation, both of which are symptoms of the condition. Due to their similarities with the body’s extracellular matrix, electrospun nanofibrous dressings are an excellent alternative. The structures have a significant surface area in comparison to their volume and they contain minute pores that make it easier for cells to adhere to one another, multiply and exchange nutrients. It is common practice to combine natural polymers, such as collagen, chitosan, and gelatin, with synthetic polymers, such as polycaprolactone or polyethylene glycol, to improve their performance (Kotteeswaran et al., 2024). The ability of nanofibers to transport bioactive substances, including antibiotics, growth hormones, and stem cells, to specific areas is remarkable. This ability enables nanofibers to maintain the continued presence of these chemicals over extended periods. These treatments have been demonstrated to be highly effective in the management of chronic wounds, burns, and diabetic ulcers, because they improve moisture retention, induce angiogenesis, and reduce edema (Jonidi Shariatzadeh et al., 2025). According to recent research, incorporating biosensors into nanofibrous mats may enable real-time assessment of moisture and pH levels in surrounding wound environments. Our perspective on individualized wound therapy might shift significantly. As a result of the development of these macromolecular dressings, the use and versatility of organic polymers have increased significantly. The ability of these materials to convert into hydrogels, films, foams, sponges, and nanofibers, each of which offers specific functional advantages, has enabled the development of novel, biologically sensitive, and patient-centered methods for the treatment of wounds (Hiwrale et al., 2023; Qu et al., 2024). Newer, more adaptive methods of wound healing are replacing traditional methods of closure; therefore, researchers and physicians are focusing on advanced dressing systems that leverage the natural bioactivity and flexibility of macromolecular compounds, which are used in many of these systems. An exciting new type of advanced wound dressing technology is cryogel-based wound dressings. Cryogels are rapidly gaining significant interest in both the scientific and medical communities because of their ability to provide unique healing capabilities for injured tissues, resulting from the porosity generated during cryopolymerization (the manufacture of cryogels at cryogenic temperatures). The pores facilitate the efficient flow of oxygen and nutrients, while also promoting rapid fluid absorption in wounds. This material is distinct from other foams or hydrogels. Cryogels can facilitate gas exchange, exhibit significant mechanical strength, and deliver pharmaceuticals to targeted areas of chronic or severe wounds. Natural polymers such as hyaluronic acid, chitosan, gelatin, and alginate can be used independently or in combination with synthetic polymers to produce cryogels. The cryogels exhibit varying degrees of flexibility and structural integrity. The device is sufficiently robust to securely cover the opening (Amonkol and Choochottiros, 2026). At the same time, its open structure facilitates the gradual release of growth factors, antibiotics, or stem cells contained within. Cryogels can be injected into wounds and fill complex voids without leaving behind space. They may deform during the time it takes them to harden; however, due to their unique properties and very large size, cryogels can be used to incorporate biosensors and/or pH-responsive devices into wounds, enabling precise treatment. The future of macromolecular wound therapies would be advanced by using cryogels primarily in non-healing and slow-healing wounds (Liu et al., 2024). An overview of commonly used synthetic and natural biomaterials in wound dressings, along with their main clinical uses and functional attributes, is summarized in **Table 7**.

**Table 7:** Biomaterials and their characteristic use in wound healing.

Sl.No.	Biomaterial	Use	Characteristics	References
Synthetic biomaterials				
1.	Polymeric Films	Coverings of surgical incisions	Establish a moist environment for the wound	(Oliveira et al., 2023)
2.	Foams	Moderate to heavily exudative wounds	Prevents bacterial infection and dehydration	(Gou et al., 2024)
3.	Hydrocolloids	Wounds with low to moderate exudate, necrotic wounds, minor burns, and pressure injuries. It cannot be used for infected wounds.	Maintains a hypoxic environment to aid in autolytic debridement	(Tarazi and Bitterman, 2023)
4.	PLGA	Fabrication of skin substitutes	Enhances neovascularization	(Pan et al., 2022)
5.	Polyethylene glycol (PEG)	Diabetic sores	Aid in collagen deposition and supplement cell proliferation in the wound bed	(Tang et al., 2022)
6.	Polyurethane (PU)	Semi-permeable film or foam dressings for burns, ulcers, and surgical wounds maintain moisture, absorb exudate, and permit oxygen exchange.	Protects against bacterial invasion of wounds; particularly useful for wounds that heal best in a moist environment.	(Shen et al., 2022)
7.	Polycaprolactone (PCL)	Acute and chronic wounds	Activates the integrin-1 signaling pathway to initiate fibroblast proliferation	(Osanloo et al., 2024)
Natural biomaterials				
1.	Alginate	Bleeding wounds after surgery, ulcers on the legs	Has hemostatic capabilities	(Mazurek et al., 2025)
2.	Silk Sericin	Second-degree burn wounds	Promotes increased fibroblast proliferation, which in turn increases wound-site cell density and collagen synthesis.	(Wang et al., 2024)
3.	Acetobacter xylinum	Dermabrasions, skin lesions, persistent ulcers, basal cell carcinoma/skin transplant, severe body burns, face peeling, sutures	Protects from pathogenic agents	(Han et al., 2024)
4.	Fucoidan	Dermal Burns	Promotes fibroblast migration and stimulation at the injury site; promotes tissue re-epithelialization	(Dores et al., 2025; Pei et al., 2025)
5.	Cellulose	Burns, ulcers	Prevents microbial infection at the wound site	(Meng et al., 2023; Dang et al., 2025)
6.	Pectin	Infected wounds, Burn wounds	Shows anti-inflammatory action	(Aliyeva et al., 2024)
7.	Gelatin	Diabetic Wounds	Hastens the formation of granulation tissue; shows hemostatic and anti-thrombogenic properties	(Garabet et al., 2023)
8.	Carrageenan	Hemorrhagic wounds, bone and cartilage regeneration	Antiviral properties	(Neamtu et al., 2022; Das and Bal, 2024)
9.	Pullulan	Chronic wounds and burns	Anti-coagulant, anti-inflammatory	(Elangwe et al., 2023)
10.	Collagen	Surgical repair	Promotes cell proliferation, migration, and differentiation	(Jeon et al., 2025)
11.	Chitin	Diabetic wounds	Increase the release of platelet-derived growth factor-AB and transforming growth factor-1	(Zheng et al., 2023)

Recent advances in our understanding of the cellular events that occur during the different phases of wound repair have led to a better understanding of how biomaterials interact with the molecular signaling pathways that drive tissue repair. For example, the PI3K/AKT and MAPK/ERK pathways have been identified as key signaling pathways that stimulate keratinocyte proliferation and facilitate fibroblast migration; therefore, the activity of either pathway can hasten wound closure. The use of hyaluronic acid and collagen as scaffolds also maintains growth factor activity and activates signals that result in AKT-mediated cell survival in dermal fibroblasts (Duan et al., 2024). Similarly, the TGF-β/Smad pathway orchestrates extracellular matrix deposition and scar formation, where silk fibroin and gelatin hydrogels have demonstrated modulatory effects by attenuating excessive Smad2/3 signaling, thereby reducing hypertrophic scarring (Jin et al., 2025). Angiogenesis, a hallmark of the proliferative phase, is tightly regulated by VEGF and hypoxia-inducible factor-1α (HIF-1α). Bioactive metal-ion-coordinated biomaterials, such as Zn²^+^- or Cu²^+^-modified hydrogels, enhance VEGF secretion and stabilize HIF-1α, thereby promoting neovascularization in ischemic wounds. During remodeling, matrix metalloproteinases (MMPs) and their inhibitors (TIMPs) balance collagen degradation and deposition. Chitosan-based dressings and nanoparticle composites have been reported to downregulate excessive MMP-9 activity, creating a favorable matrix environment for long-term tissue repair (Yang et al., 2026). Recent research shows that wound healing is more complex than a series of histological changes; it is also an intricate molecular conversation. The result will be future advancements in biomaterials that can alter the wound healing process through their design. Wound dressings will evolve from purely supportive mechanisms to active partners in the healing process by matching scaffold chemistry, ion coordination, and bioactive load with specific molecular targets.

## 7 Functional modifications and bioactive integration of natural macromolecular dressings

Over time, natural macromolecular wound dressings have evolved from simply being connective tissue coverings to active therapeutic platforms through the addition of functional modifications and bioactive components. This modification enables faster wound-healing rates, along with specific antibacterial, anti-inflammatory, and pro-regenerative properties, making these products more effective in their interactions with the surrounding wound environment (Zhang et al., 2025). To address the complications associated with chronic and infected wounds, bioactive compounds such as cytokines, plant extracts, growth factors, and antimicrobial nanoparticles are often used in these dressings. Many people use silver nanoparticles because they can eradicate a variety of bacteria. Silver nanoparticles are effective in eradicating bacteria because they disrupt the bacterial cell wall and prevent the replication of bacterial DNA by generating reactive oxygen species (Nasra et al., 2024). When we add them to natural polymer networks such as gelatin, chitosan, or alginate, they work better with living organisms and last longer. Researchers have also studied how zinc oxide and copper oxide nanoparticles can fight bacteria and inflammation. These changes are crucial for healing chronic wounds because they prevent bacteria and biofilms from growing (Ali et al., 2025). A recent study has improved the performance and safety of the polymer matrix by adjusting the particle size and distribution within it. The treatment will be both safe and effective. Contemporary macromolecular therapies employ both inorganic compounds and bioactive molecules such as PDGF, TGF-β, and VEGF, all of which are growth factors. The elements are required to modify the extracellular matrix, recruit fibroblasts, and establish the vascular endothelium. Hydrogels, nanofibers, and sponges can prevent these substances from reaching the wrong regions, help tissues heal faster, and keep them from contacting other parts of the body. Maintaining the stability of these proteins during fabrication and storage remains challenging (Mahnoor et al., 2025). Liposomes, microspheress and PEGylation techniques are some of the protective carriers employed for this. Adding phytochemicals and natural extracts to wound dressings has strengthened their ability to fight pathogens, inflammation, and free radicals (Sanati and Amin Yavari, 2024). Researchers have studied extracts of curcumin, quercetin, aloe vera and centella asiatica extensively because they may modify how scars respond to oxidative stress and inflammation. When these medications are combined with macromolecular scaffolds, they help chronic and infected wounds heal faster and improve the performance of the original material (Fernandes et al., 2023). Antioxidants and essential oils have also been added to kill a wide range of microorganisms and prevent the development of antibiotic resistance. The best and most advanced way to care for wounds is to use smart, flexible dressings. Things in the environment, like pH, temperature, glucose levels, and enzyme activity, can change their properties. When pH-sensitive hydrogels come into contact with an alkaline wound, they alter shape and release hormones or antimicrobials (Nezhad-Mokhtari et al., 2024). These devices can help doctors make treatment decisions, provide diagnostic information, and support immediate wound care. The newest biosensors can provide real-time monitoring of temperature, pH, and moisture levels, allowing physicians to assess infection risk and determine whether recovery is taking too long. There is also functional modification *via* changes to the polymer backbone’s chemical structure. Functionalizing drugs by adding functional groups such as carboxyl, amine, or hydroxyl moieties will enhance their ability to bind covalently with tissue, retain water, and bind to tissue. Cross-linking agents such as genipin and glutaraldehyde, or an enzymatic system, can adjust the drug release patterns, mechanical strength, and the rate of drug degradation (Manoharan Nair Sudha Kumari and Thankappan Suryabai, 2024; Vo and Trinh, 2025). We can change the scaffold structure to include micro- and nanoscale features that help cells grow, differentiate, and align. Moreover, we can do these using methods such as electrospinning, 3D printing, and freeze-drying (Wang et al., 2022). The overall performance of base dressings has significantly increased due to the addition of organic macromolecules such as peptide-based and polysacharide based materials. For example, heparin and hyaluronic acid (such as Dermagraft™) (Hart et al., 2012) facilitate vasodilation and reduce the amount of edema present in the local blood vessels of the area around a wound. RGD peptide sequences enhance cohesive cell interactions and promote cell motility. The presence of these chemicals in the wound bed serves as a means for enhancing the normal healing process of the body by serving as chemical messengers for tissue repair. In conjunction with peptides, these biomaterials also serve to reduce the time for complete healing of full-thickness wounds and improve the aesthetic results after healing. Research has demonstrated that the inclusion of bioactive molecules and alteration in their behavior during the healing process greatly enhances the effectiveness of natural macromolecular treatments. Technologies that utilize this type of approach will enable the creation of personalized wound management systems that are tailored to the individual needs of each patient. The construction of these systems is not only designed to protect but also to enhance the healing process. As clinical research evolves, these adaptable and innovative dressing systems will introduce a new paradigm for delivering individualized wound care (Zielińska et al., 2023).

## 8 Clinical and preclinical performance evaluation of natural macromolecular dressings

**Figure 8:**
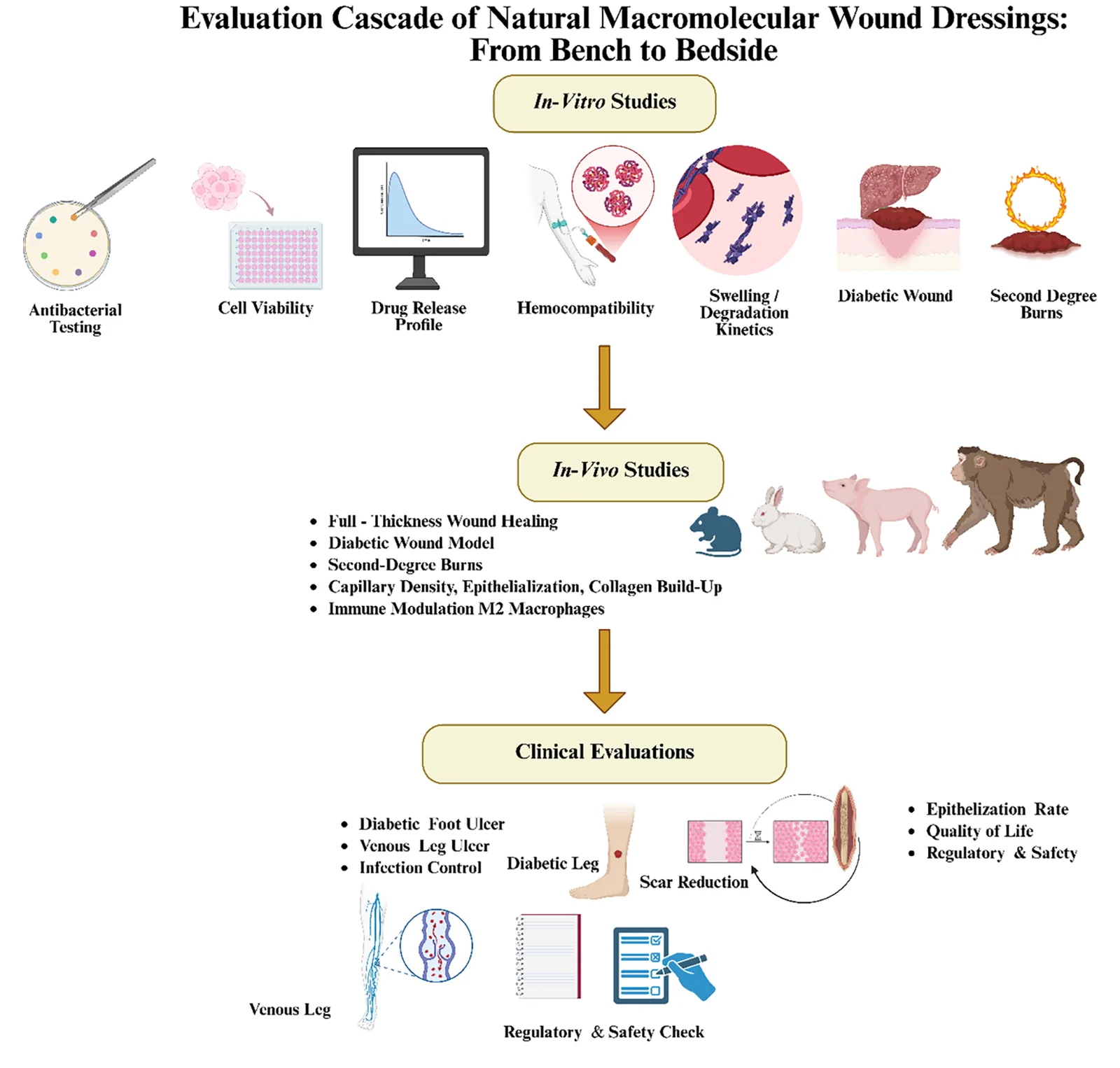
Translational evaluation cascade of natural macromolecular wound dressings illustrating progression from in vitro characterization to in vivo validation and clinical assessment. Initial laboratory investigations include antibacterial activity, cytocompatibility, hemocompatibility, drug release profiling, and swelling or degradation kinetics. Subsequent in vivo studies in relevant animal wound models evaluate parameters such as re-epithelialization, angiogenesis, collagen deposition, and immune modulation. Clinical evaluation focuses on therapeutic performance in conditions such as diabetic foot ulcers and venous leg wounds, along with assessment of infection control, scar reduction, quality-of-life outcomes, and regulatory safety considerations. This multistage framework supports the systematic translation of biomaterial-based wound dressing technologies from experimental development to clinical implementation.

Several *in vitro* and *in vivo* studies, as well as various promising clinical trials, have suggested that natural macromolecular wound dressings can be helpful. These tests are necessary to determine how useful, safe, and effective different biomaterials are for treating wounds. A stepwise evaluation pathway from *in vitro* testing to *in vivo* studies and clinical trials for natural macromolecular dressings is depicted in **Figure 8**. *In vitro* studies conducted in labs mainly focus on testing how well an antibiotic works, how well it interacts with cells and blood, how well it swells, how well it breaks down in the environment, and how well it releases medication in a controlled way. These factors provide a basic idea of how materials interact with living things (Ezike et al., 2023; Ewii et al., 2025). Chitosan-based composites are highly effective at killing both Gram-positive and Gram-negative bacteria. They also help fibroblasts proliferate and produce collagen under controlled conditions (Pothal et al., 2023). Similarly, alginate and collagen matrices have been shown to promote the adhesion of keratinocytes, angiogenesis, and cellular infiltration *in vitro* (Hosty et al., 2024). Researchers frequently employ mice, pigs, or rabbits with certain sorts of wounds, like full-thickness excisions, diabetic ulcers, or burn injuries, to test new drugs or treatments before they are tested on people. These models help us learn more about how wounds contract, how new skin cells grow, how granulation tissue forms, how blood vessels form, and how inflammatory cells enter the wound (Mukherjee et al., 2022; Ghanbari et al., 2024). In mouse excision models, alginate-based dressings have been shown to accelerate wound healing, reduce neutrophil infiltration, and increase capillary density. Chitosan-collagen sponges soaked in antibiotics or growth hormones have helped wounds in diabetic models heal faster and have fewer bacteria (Yang et al., 2020). Recent *in vivo* studies show that biomaterials can modulate the immune response, particularly by promoting macrophages toward the M2 phenotype, which supports healing rather than persistent inflammation (Jackson et al., 2025). In preclinical trials, silk sericin and bacterial cellulose dressings have shown great promise. Silk sericin dressings have helped wounds heal faster and promoted collagen formation in models of second-degree burns (Borges et al., 2024). Bacterial cellulose matrices create a moist environment that encourages the formation of new blood vessels and skin cell proliferation *via* the promotion of angiogenesis and epidermal cell proliferation, respectively. In addition to providing a barrier to the external environment, there is increasing evidence that these natural biomaterials aid in healing. While clinical data are limited, initial clinical trials support findings from preclinical studies. For example, clinical studies have demonstrated that when alginate dressings were used on patients with venous leg ulcers, surgical wounds, and pressure ulcers, their wounds healed faster than with conventional gauze-based dressings; furthermore, patients experienced greater comfort and improved exudate management than those treated with conventional dressings (Utoiu et al., 2024). Chitosan-based dressings have shown significant efficacy in reducing wound area and infection rates in diabetic foot ulcers and trauma-linked injuries. In a randomized clinical evaluation, collagen-based dressings significantly improved the epithelialization index. They reduced healing time in full-thickness surgical wounds and chronic ulcers (Yang et al., 2023). Moreover, multifunctional hydrogels containing macromolecular blends with silver nanoparticles, growth factors, or anti-inflammatory compounds have demonstrated favorable outcomes in pain relief, infection prevention, and long-term scar reduction in pilot-scale human studies (Popescu et al., 2023). Evaluation methods are continually evolving, using new imaging techniques, scoring scales for wounds, and molecular tests that measure healing through biomarkers, cell movement, and the deposition of the ECM. In addition to histology and tensile-strength testing, cytokine profiling has become essential in preclinical and clinical studies of wound healing. Typical parameters reported in studies include the percentage of re-epithelialization, vascular density, granulation tissue thickness, and degree of inflammation. These provide quantitative evidence supporting the regenerative potential of materials. While the results to date have been promising, challenges remain in standardizing the animal models used to evaluate wound healing, ensuring the reproducibility of the models, and translating laboratory data into large-scale clinical evidence. Variations in patient characteristics, health histories, wound types, and treatment approaches frequently make it difficult to achieve a uniform understanding of clinical outcomes. Moreover, securing regulatory approval for wound dressings composed of biomaterials necessitates extensive evidence of long-term safety and rigorous clinical validation. In laboratory and clinical studies spanning many years, it has been demonstrated that macromolecule-based dressings are extremely beneficial for promoting the healing of wounds of all types, especially complex or chronic wounds. Macromolecule-based dressings can promote wound healing through different mechanisms of action, including preventing infection, reducing inflammation, and stimulating tissue regeneration. However, further research and clinical studies are currently underway to provide evidence of the viability of these dressings in wound care practices. In general, macromolecule-based dressings are an attractive option for providing safe and effective wound-healing solutions that meet patients’ actual healing needs (Alberts et al., 2025a).

Macromolecular dressings have evolved from simply acting as passive building blocks to becoming active participants in the bioregulation of a wound bed. This change in how macromolecular dressings are developed means that the materials used to create these dressings can now affect wound biology by interacting with cells and biological pathways within the wound bed to improve wound healing rather than simply acting as a barrier to the injury itself. Cho and co-workers (Cho et al., 2026) provided compelling evidence that polysaccharide-protein hybrid interfaces with amphiphilic properties can influence cytokine expression, promote keratinocyte migration, and ultimately accelerate epithelial closure. Such findings exemplify how functional materials are being engineered to actively engage with the wound microenvironment, pointing to a new generation of therapeutic dressings that integrate structural support with the molecular regulation (Pan et al., 2025). Zheng and colleagues demonstrated that collagen-polysaccharide matrices enriched with metal nanoparticles enhanced vascular endothelial growth factor (VEGF) activity while also limiting bacterial growth at the wound surface. These dual functions highlight the potential of combining biopolymer scaffolds with inorganic components to create multifunctional dressings that support both tissue regeneration and antimicrobial defense (Zheng et al., 2024). Advancements in technology indicate a move away from traditional dressings toward multifunctional composite dressings that not only enhance the dressing’s wound-healing properties but also provide wound-healing benefits. A closer look at the various types of dressing indicates numerous differences. At the same time, protein-based scaffolds offer the highest tensile strength and bioactive signaling properties, while polysaccharide-based dressings primarily provide moisture management and hemostatic properties. Hybrid dressings will combine the advantages of both types. However, hybrid-type dressings have the potential for limited long-term mechanical support and may require chemical or structural reinforcement. As indicated by recent studies, the ionic coordination of zinc ions and copper ions, along with the use of nanocomposite-type dressings, may provide an additional wound specific advantage (Liu et al., 2025). For example, protein-rich constructs are more effective for mechanically stressed surgical wounds, polysaccharide-based hydrogels are optimal for exudative ulcers, and hybrid nanocomposites are particularly effective in treating infected chronic wounds. This comparative framework not only overcomes existing gaps in descriptive reviews but also provides a decision-making approach that strengthens the clinical translation of macromolecular biomaterials. Despite promising outcomes, the lack of standardized clinical protocols and variability in patient populations remain key barriers to the reproducibility and large-scale validation of these biomaterial systems.

A plethora of clinical studies have demonstrated the effectiveness of organic macromolecular dressings (e.g., alginate dressings) in reducing healing time for venous leg ulcers (Norman et al., 2018). Likewise, collagen dressings not only help expedite the healing of chronic wounds by stimulating the formation of new tissue and skin layers but also significantly reduce diabetic foot ulcer infection recurrences when used with chitosan dressings. This provides biological and clinical evidence of credibility for any biological and clinical evidence supporting protein- or polysaccharide-sed systems in modern wound management (Jin et al., 2026).

## 9 Environmental sustainability and functional efficiency of polysaccharide based dressings

Natural polysaccharides (such as alginate, chitosan and hyaluronic acid) are great materials to use as biomaterials because they provide a number of beneficial biological properties and also are much more sustainable than the traditional non-renewable synthetic (petrochemical) wound dressings used today. These biodegradable materials come from naturally-replenishing sources and will have a lower profile in the environment after disposable than traditional synthetic (petrochemical) products do due to their degradation properties. In a physiological environment, alginate will degrade through ion exchange, chitosan through lysozyme-mediated enzymatic pathways, and hyaluronic acid will degrade through endogenous enzymatic processes (as it is a natural part of the extracellular matrix). The byproducts produced from each of these degradation processes are not toxic, so there will be very little accumulation of them in the environment over a long period of time. The functional properties of polysaccharides provide indirect residue-free advantages through their ability to confer environmental benefits beyond biodegradability, such as moisture retention, tissue adhesion, and the promotion of healing/restoration of the physical structure. Because these functionalities reduce the number of times dressings must be replaced, there will be a subsequent reduction in the amount of material used and in the amount of medical waste generated from dressing changes (Sharma et al., 2026).

Additionally, both the antimicrobial and clotting properties of polysaccharides (for example, chitosan) will reduce the amount of chemical agents needed to treat wounds, thereby further reducing the materials required for wound treatment. When considering the process used to create polysaccharide-based systems, polysaccharides can be manufactured using less harsh, less environmentally harmful techniques than synthetic polymers. Ionic crosslinking, gelation, and solvent-free or aqueous solution techniques are commonly used to produce polysaccharide-based topical systems, thereby reducing the need for harsh chemicals or energy-intensive manufacturing processes, ultimately resulting in a product that demonstrates principles of environmental chemistry and supports the creation of safer, more environmentally sustainable biomedical products. These manufacturing processes are in direct opposition to many synthetic polymeric dressings, which are manufactured through complex chemical processing routes using hazardous chemicals and create waste that will not decompose in the environment (B et al., 2024; Jin, 2026).

Polysaccharides offer many environmental benefits when viewed from a broader lifecycle perspective. Their natural sources are abundant, such as algal (marine) sources through alginate; chitin from crustacean shells; and hyaluronic acid from microbes or other animals, indicating their ability to be renewable and sustainable. Polysaccharides are also biodegradable, so once used clinically in the wound-healing process, they break down in the body or the environment over time. They require more time to decompose than their non-biodegradable synthetic counterparts, and they exhibit prolonged environmental persistence. Thus, designing biodegradable materials with the ability to heal wounds effectively serves a dual purpose: 1) by providing performance clinically (wound healing) and 2) by being environmentally responsible. Ultimately, the relationship among the biodegradation (decomposition) of the material, their ability to perform clinical functions, and the environmentally sound way they are processed suggests that polysaccharide-based dressings offer a promising opportunity for future sustainable healthcare solutions. Future research on polysaccharides will determine the environmental benefits of these materials through lifecycle analysis, degradation kinetics, and waste reduction, enabling a more complete assessment of their utility in developing environmentally sound wound-management programs (Liu et al., 2024; Porwal et al., 2025).

**Table 8** provides a comparative analysis of polysaccharide-based biomaterials versus traditional synthetic dressings. The analysis shows that there are many differences between the two types of dressings in their biodegradability, biological degradation mechanisms, waste production, and sustainability. These differences in performance indicate the benefits that may be derived from using natural macromolecular systems to help alleviate environmental problems without reducing or improving their therapeutic value.

**Table 8:** Comparison of environmental sustainability and functional efficiency between synthetic and polysaccharide-based wound dressings.

Parameter	Synthetic wound dressings	Polysaccharide-based wound dressings	References
Source	Petrochemical-derived polymers	Natural and renewable sources	(Satchanska et al., 2024)
Biodegradability	Low or non-biodegradable	High biodegradability	(Lee et al., 2023; Liang et al., 2023)
Degradation mechanism	Slow, non-biological degradation	Enzymatic and physiological degradation	(Dutta and Sit, 2024)
Environmental persistence	Long-term accumulation	Minimal persistence after degradation	(Ren et al., 2022)
Waste generation	High due to frequent replacement	Reduced due to improved healing efficiency	(Moradifar et al., 2025)
Dressing change frequency	Frequent	Reduced frequency	(Cui et al., 2022)
Fabrication process	Chemical synthesis, solvent-based	Green processing, aqueous/ionic methods	(Gupta et al., 2025)
Toxicity risk	Possible residual toxicity	Generally low and biocompatible	(Aderibigbe, 2022)
Sustainability profile	Limited	High sustainability potential	(Behrooznia and Nourmohammadi, 2024)

As shown in **Table 8**, the advantages of using polysaccharide-based wound dressings include their biodegradability, reduced persistence in the environment, and better material use than non-biodegradable dressings. All of these factors make them good sustainable substitutes for traditional synthetic wound dressings and help alleviate growing concerns about biomedical waste.

### 9.1 Sustainability and life cycle considerations

Increased clinical use of polysaccharide-based dressings enhances interest in their environmental sustainability in terms of their entire product life cycle. Natural polysaccharides such as alginate, chitosan, bacterial cellulose and hyaluronic acid differ from synthetic polymers because they are derived from sources that are renewable biological resources such as marine biomass, seashell waste, fermentation by microorganisms and plants. Their application would help save the world from growing wastes and reduce reliance on fossil-based materials. As far as production is concerned, polysaccharide dressings may have an environmental impact due to extraction, purification, chemical modification;. and fabrication procedures. Traditional extraction methods may involve extensive use of solvents like water, acids, bases or organic solvents, whereas environmentally friendly methods like enzyme extraction, fermentation and liberal use of water in gel fabrication have proved to use less chemicals and produce less waste. More recent life cycle assessment (LCA) studies show that the choice of materials, energy demand during sterilization, drying and transportation play a significant role in determining the environmental impact of biomaterials. An additional advantage of polysaccharide dressings is their biodegradability under physiological or environmental conditions, reducing the long-term persistence of medical waste compared with many conventional synthetic polymers (Salvay, 2025; Khachatryan et al., 2026). However, environmental performance depends not only on the polymer source but also on crosslinking chemistry, incorporated metal ions, nanomaterials, sterilization procedures, packaging and end-of-life disposal. Therefore, future development of metal coordinated polysaccharide dressings should integrate sustainable material sourcing, green manufacturing technologies and standardized life-cycle assessment alongside biological activity as well as clinical efficacy to support environmentally responsible translation into healthcare.

## 10 Integrated design framework for polysaccharide based wound dressings

Despite substantial advances in polysaccharide-based biomaterials, a unified strategy linking material composition, structural design, and stage-specific biological functionality remains inadequately defined. To address this gap, a mechanism-driven design framework is proposed to guide the development of advanced wound dressings.

### 10.1 Material composition and chemical functionality

Wound dressing properties (physicochemical and biological) are determined by polysaccharides like alginate, chitosan, and hyaluronic acid. Through ionic gelation, alginate is able to manage exudate. Chitosan’s cationic properties make it useful for providing antibacterial and/or hemostatic activity. Hyaluronic acid has been shown to be important in regulating cell migration and remodeling the extracellular matrix. Functional enhancements can be achieved through the addition of proteins (collagen, gelatin, silk fibroin), metal ions (Zn²^+^, Cu²^+^, Ag^+^), or nanoparticles to modulate angiogenesis, antibacterial agents, and therapeutic release control.

### 10.2 Scaffold architecture and functional engineering

The various types of scaffold architecture (hydrogels, nanofibers, sponges, films, and cryogels) determine many important properties, including porosity, mechanical strength, oxygen permeability, and fluid absorption. The ability to manipulate structural and functional properties of scaffolds with engineering techniques (ionic crosslinking, metal-ligand coordination, electrospinning, and 3D bioprinting) gives researchers great control over scaffolds. Furthermore, the use of stimuli-responsive systems allows for dynamic and spatiotemporally controlled delivery of drugs to the wound microenvironment.

### 10.3 Biological targeting and stage-specific functionality

**Figure 9:**
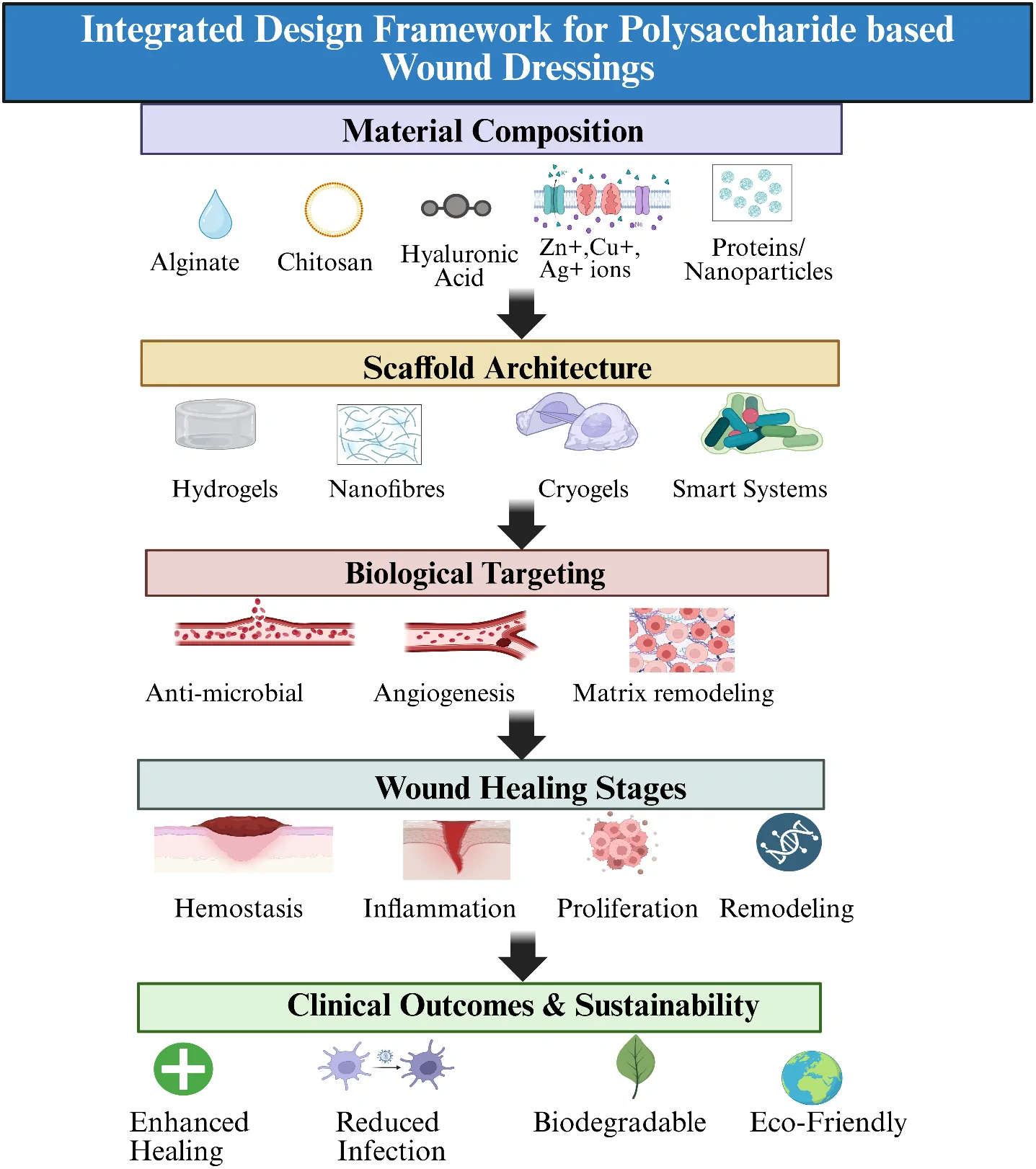
This schematic illustrates the hierarchical relationship between material composition (alginate, chitosan, hyaluronic acid, and bioactive additives), scaffold architecture (hydrogels, nanofibers, cryogels, and smart systems), and biological targeting mechanisms (hemostasis, antimicrobial activity, angiogenesis, and matrix remodeling). These elements are aligned with the sequential stages of wound healing (hemostasis, inflammation, proliferation, and remodeling) to achieve improved clinical outcomes, including enhanced tissue regeneration, reduced infection, and environmentally sustainable performance. The framework emphasizes a mechanism-driven approach for the rational design of next-generation wound dressings, and highlights the direct correlation between material design parameters and biological performance.

When creating an effective wound dressing it is important to consider the dynamic phases of wound healing in order to properly support healing. During the early phases of wound healing hemostatic and antimicrobial function is required while angiogenesis and tissue regeneration are dominant processes during the proliferative phase of healing. In the remodel phase regulatory control over collagen deposition and fibrotic tissue formation is important. Additionally, through targeting important signaling pathways such as PI3K/AKT, VEGF/HIF-1α and TGF-β/Smad biomaterials can regulate cellular responses to improve the overall outcome of healing. The proposed integrated design framework is illustrated in **Figure 9**, highlighting the interrelationships among material composition, scaffold architecture, biological targeting, wound-healing stages, and clinical outcomes.

## 11 Limitations, challenges, and translational barriers

Although there has been substantial advancement in the development and use of polysaccharide-based biomaterials for the purpose of healing chronic wounds, there are still many limitations that will need to be addressed in order for these materials to be successfully used in a clinical setting. One of the most significant challenges is that the variability between batches of naturally derived polymers, such as chitosan, alginate, and collagen, can be very high. The result of this difficulty in producing consistently sized and manufactured materials means that variations in the molecular weight, degradation degree of chitosan, and impurities that are dependent on the source will result in thesis articles having very different physicochemical and biological properties in terms of their *in vitro* performance, reproducibility, and ability to meet the requirements for regulatory approval. Additionally, there is concern regarding the lack of mechanical strength of many polysaccharide-based wound dressing materials. Although hydrogels and porous scaffolds will provide excellent moisture retention and biocompatibility, the lack of mechanical stability in these materials often means that chemical crosslinkers will need to be applied and/or they must be combined with synthetic polymer mixtures to achieve an acceptable level of mechanical stability; this could result in losing the ability to biodegrade or introduce potential toxicity. The delivery of bioactive compounds, such as growth factors, such that there is a constant and reliable supply to the wound site is currently very challenging because they can be very unstable and readily degrade in physiological solutions. Finally, there is concern about the long-term biosafety of nanoparticles and metal ions used within polysaccharide-based dressings, particularly those that are incorporated for their antimicrobial and angiogenic properties, due to the fact that the prolonged contact time associated with their use in chronic wound applications creates a greater risk of causing cytotoxicity.

Most studies to date have only focused on small animal models or laboratory (*in vitro*) studies. There is an absence of large-scale, well-controlled clinical trials to date. In addition, there are many differences in wound types, variability among patients, and also co-morbidities that challenge the ability to translate pre-clinical findings to a clinical setting. Further complicating the issue will be limitations in manufacturing capabilities and scalability. Although techniques such as electrospinning, 3D bioprinting, and cryogel fabrication show promise, they require specialized equipment and may not be able to be easily adapted for large-scale production. Additionally, while polysaccharide-derived materials are regarded as environmentally sustainable, there remain limited comprehensive lifecycle assessments and standardized degradation studies to date. Quantitative assessments of environmental impacts are still very limited and will need to be investigated further. In order to address the above-mentioned limitations, it will be crucial that researchers develop standardized material characterization procedures; implement better fabrication strategies; and foster interdisciplinary collaboration between material scientists, clinicians, and regulatory agencies to enable clinical translation of next-generation wound-dressing systems. Each of these limitations demonstrates the need for standardized evaluation processes and interdisciplinary collaboration to abolish the disparity between bench-side and bedside manufacturing.

## 12 Future prospects and conclusion

Natural macromolecular biomaterials (e.g., polysaccharide-based systems such as hyaluronic acid, chitosan, and alginate) continue to be recognized as vital components towards the development of next generation advanced wound-dressing technologies. As demonstrated in this comprehensive review, natural macromolecular biomaterials possess unique structural adaptability, biological compatibility, and functional tunability, which impact the molecular dynamics associated with wound healing. In fact, modern macromolecular dressings are no longer simply passive protective coverings, but instead, evolving into biointeractive therapeutic platforms capable of modulating various aspects of inflammation, promoting angiogenesis, and encouraging extracellular matrix remodeling throughout all phases of healing. Moreover, the data presented here provides compelling evidence supporting the merger of material chemistry with biological signaling pathways. By using combinations of scaffold composition, ionic crosslinking techniques, and biomolecular delivery systems to provide precise modulation of various biological activities (e.g., fibroblast proliferation, keratinocyte migration, and immune cell polarization), ultimately, these scientists are able to create innovative new approaches to enhancing wound care. These design factors are especially important when dealing with complex wound conditions characterized by low oxygen levels, high bacterial counts, excess fluid, or poor blood flow. In this case, hybrid protein-polysaccharide systems and metal-ion-coordinated biomaterials show great promise for achieving multifunctionality while maintaining structural stability and controlling drug release. Even with promising results from early studies and trials, there are still obstacles to overcome in making these materials a standard part of clinical practice. Concerns about large-scale reproducibility, long-term mechanical durability, regulatory approval, and patient-specific variability continue to affect the effective use of next-generation dressing technologies. To achieve Clinical Translation and promote the widespread adoption of biomaterial systems, testing protocols must be standardized, and regulatory and environmental guidelines must be integrated. Future research should develop material selection criteria that logically combine scaffold architecture, bioactive methods, the type of wound present and its healing stage, and the local microenvironmental signals specific to the patient. Furthermore, future studies should include metrics for quantitatively evaluating the environmental impacts of polysaccharide-based wound dressings, using standardized measures such as biodegradation rate, waste reduction efficiency, and lifecycle assessment. Novel technologies, such as biosensor-integrated dressing platforms, stimuli-responsive hydrogels, and advanced manufacturing methods like electrospinning and bioprinting, are expected to make wound care even more precise and customized. In parallel, greater emphasis on biodegradable material selection, green fabrication strategies, and responsible disposal practices will be critical to reducing the environmental footprint of wound care technologies while enabling scalable clinical implementation. The integration of polysaccharide chemistry and coordinated-driven framework engineering or mechanism guided design will change the way we will manage wounds in the future. In the future, most polymeric-based materials will be developed as programmable regenerative interfaces that aid in driving tissue regeneration instead of only relying on our previous experiences to choose which materials we will use to facilitate tissue repair. In addition, the future polymeric-based products will incorporate both therapeutic outcomes and sustainable practices in their overall design, thus contributing to the future adaptive regenerative systems that enhance clinical outcome while promoting environmentally sound health practices. This review provides a mechanism driven approach to biomaterial design by illustrating the design of polysaccharide-based products as an integrated means of providing biological functions, and developing a rationale for the design of the next generation of wound dressings. Future advances in this area will depend on the development of reproducible, scalable, and clinically valid products to allow for successful translation into clinical practice.
